# Retrieval augmented scientific claim verification

**DOI:** 10.1093/jamiaopen/ooae021

**Published:** 2024-02-21

**Authors:** Hao Liu, Ali Soroush, Jordan G Nestor, Elizabeth Park, Betina Idnay, Yilu Fang, Jane Pan, Stan Liao, Marguerite Bernard, Yifan Peng, Chunhua Weng

**Affiliations:** School of Computing, Montclair State University, Montclair, NJ 07043, United States; Department of Medicine, Columbia University, New York, NY 10027, United States; Department of Medicine, Columbia University, New York, NY 10027, United States; Department of Medicine, Columbia University, New York, NY 10027, United States; Department of Biomedical Informatics, Columbia University, New York, NY 10027, United States; Department of Biomedical Informatics, Columbia University, New York, NY 10027, United States; Department of Applied Physics and Applied Mathematics, Columbia University, New York, NY 10027, United States; Department of Applied Physics and Applied Mathematics, Columbia University, New York, NY 10027, United States; Institute of Human Nutrition, Columbia University, New York, NY 10027, United States; Department of Population Health Sciences, Weill Cornell Medicine, New York, NY 10065, United States; Department of Biomedical Informatics, Columbia University, New York, NY 10027, United States

**Keywords:** natural language processing, clinical trial, evidence retrieval, deep learning, evidence appraisal

## Abstract

**Objective:**

To automate scientific claim verification using PubMed abstracts.

**Materials and Methods:**

We developed CliVER, an end-to-end scientific **Cl**a**i**m **VER**ification system that leverages retrieval-augmented techniques to automatically retrieve relevant clinical trial abstracts, extract pertinent sentences, and use the PICO framework to support or refute a scientific claim. We also created an ensemble of three state-of-the-art deep learning models to classify rationale of support, refute, and neutral. We then constructed CoVERt, a new **CO**VID **VER**ifica**t**ion dataset comprising 15 PICO-encoded drug claims accompanied by 96 manually selected and labeled clinical trial abstracts that either support or refute each claim. We used CoVERt and SciFact (a public scientific claim verification dataset) to assess CliVER’s performance in predicting labels. Finally, we compared CliVER to clinicians in the verification of 19 claims from 6 disease domains, using 189 648 PubMed abstracts extracted from January 2010 to October 2021.

**Results:**

In the evaluation of label prediction accuracy on CoVERt, CliVER achieved a notable F1 score of 0.92, highlighting the efficacy of the retrieval-augmented models. The ensemble model outperforms each individual state-of-the-art model by an absolute increase from 3% to 11% in the F1 score. Moreover, when compared with four clinicians, CliVER achieved a precision of 79.0% for abstract retrieval, 67.4% for sentence selection, and 63.2% for label prediction, respectively.

**Conclusion:**

CliVER demonstrates its early potential to automate scientific claim verification using retrieval-augmented strategies to harness the wealth of clinical trial abstracts in PubMed. Future studies are warranted to further test its clinical utility.

## Introduction

In an era of exponential growth of clinical publication, it has been increasingly challenging to identify the most relevant articles and extract relevant information for a given scientific claim.^1^ For example, LitCovid, the primary bibliographic database to track up-to-date published research on COVID-19 and SARS-CoV-2 on PubMed, has grown to more than 300 000 relevant articles in about 2 years and continues proliferating with ∼10 000 new articles added each month.^2^ This rapid growth, coupled with unvetted pre-prints, which have been included in PubMed as of June 2021,^3^ further compounds the difficulty of both evidence screening^4^ and evidence appraisal since evidence from pre-print publications can be more challenging to appraise than papers with formal peer review. As new evidence emerges, scientific understandings of many topics constantly evolve. For instance, Ioannidis et al. found that nearly one-third of highly cited clinical studies are eventually refuted as subsequent studies reach contradictions or find the original claim overstated.^5^ Without efficient information retrieval techniques, the conventional clinical evidence retrieval process entails laborious manual searches and meticulous selection of relevant studies from the vast literature, often risking missing relevant studies or mistakenly including irrelevant articles.

It is promising to mitigate these problems by implementing an automated framework that efficiently retrieves high-quality evidence for scientific claim verification. Amongst the plethora of clinical publications, randomized controlled trials (RCTs) stand as the most reliable sources of medical evidence next to meta-analysis or systematic reviews, and are essential for both clinical question answering^6^^,^^7^ and evidence-based medicine practice.^8^^,^^9^ Therefore, here, we focus on retrieving RCT studies for biomedical scientific claim verification.

Verifying scientific claims falls under the larger domain of fact verification.^10^^,^^11^ In the biomedical domain, Wadden et al. introduced the concept of scientific claim verification with a three-step approach that includes “Document Retrieval,” “Sentence Selection,” and “Textual Entailment” modules.^12^ To facilitate the development and validation of these algorithms, the authors curated the SciFact^12^ dataset. Notably, the first “Document Retrieval” module used text similarity to retrieve the most relevant abstracts without leveraging language embeddings. Pradeep et al.^13^ applied a similar three-step framework but exploited the power of the T5 language model.^14^ However, these studies may suffer from error propagation. To overcome this, Li et al.^15^ proposed a paragraph-level, multi-task learning model and used BioSentVec to compute the cosine similarities between claim and abstract embeddings. Wadden et al.^16^ leveraged the Longformer model^17^ to cross-encode the claim, title, and abstract together as input with minimal truncations. These studies achieved impressive performance in scientific claim verification against a relatively small (<10 000) document repository. They were not evaluated in real scientific use cases where hundreds of thousands of documents are queried against. To the best of our knowledge, none of these studies focus on RCTs, which are substantially different from observational epidemiological studies (eg, cohort, case-control, and cross-sectional studies) and other topics within biomedicine (eg, laboratory-based research).

Identifying the best evidence requires massive RCTs to enable rigorous and reliable study appraisal—for example, relevance, allocation concealment, intention to treat analysis, and relevant outcomes.^18^^,^^19^ Retrieval-augmented methodologies harness the capabilities of multiple information retrieval techniques (such as document vectorization,^20^^,^^21^ semantic similarity-based retrievers,^22^^,^^23^ and similarity ranking^24^^,^^25^ mechanisms). The adoption of these techniques facilitates an efficient and accurate search through vast datasets of RCT studies, improving the identification of the most relevant evidence.

Existing claim verification datasets have a specific focus, making them suitable for applications in certain clinical domains. For instance, FEVER is a general-domain fact-checking dataset created by re-writing Wikipedia sentences into atomic claims, verified against Wikipedia articles.^11^ PUBHEALTH gathered public health claims from fact-checking websites (eg, Snopes) and verified them against news articles with gold-standard labels crafted by journalists.^26^ ManConCorpus is a manually annotated corpus containing pairs of claims and sentences from 259 abstracts associated with 24 systematic reviews on cardiovascular disease.^27^ HealthVer^28^ focused on COVID-related claims, obtained by rewriting answers to questions from TREC-COVID^29^ and validated against the CORD19 corpus.^30^ COVID-Fact scraped COVID-19 claims from a social media platform (Reddit) and verified them against linked scientific papers and documents retrieved via Google search.^31^ While such datasets are essential for developing claim verification systems, only some include information regarding study design that is key for accurate evidence appraisal. Therefore, existing datasets may not be suitable for verifying clinical-related scientific claims since they do not consider the strength of studies that produced the evidence. To improve verification accuracy in the clinical domain, we introduce a new **Co**vid **VER**ification datase**t (CoVERt).** It consists of 15 claims, 96 abstracts with “support” or “refute” labels, and a set of sentences extracted from the abstracts containing rationales for claim verification. The claims were encoded using the PICO framework (Population, Intervention, Comparison, and Outcome), a widely-used knowledge representation for clinical questions.^32^ This deliberate design choice of CoVERt facilitates seamless alignment with extracted evidence from RCTs, thereby providing a foundation for utilizing these rationales in the evaluation process.

We propose an automated PICO-based **Cl**a**i**m **VER**ification framework (**CliVER**) that employs retrieval-augmented methodologies to verify claims and identify supporting and refuting clinical evidence from the clinical trial literature. We also introduce an ensemble of three state-of-the-art systems for label prediction. The outputs of the three systems are combined using majority voting. Evaluations of two testing datasets showcase the generalizability of our framework. We observed that the ensemble of two deep language models and one prompt-based model improves the verification accuracy of each model by an absolute increase in the F1 score from 3% to 11% on CoVERt. We compared CliVER’s output for 19 scientific claims from 6 disease categories to clinicians’ manual review. Our experiment revealed that CliVER achieved high accuracy in both the Oracle and Open settings.

## Methods

### Task formulation

Given a scientific claim c, we retrieved all clinical trial literature A in PubMed relevant to c. Following previous studies,^11–13^^,^^15^ for any given publication, its agreement with the claim was represented by one of three labels: Support, Refute, and Neutral (neither support nor refute). For this study, we evaluated the PubMed title and abstract for publications either with “randomized controlled trial” labeled as publication type or as Medical Subject Headings (MeSH) term. In addition, for each abstract a∈A, we retrieved rationale sentences S∈a that justify each relationship. It is worth noting that, to simplify the rational annotation and extraction, our gold standard annotations contained a single-sentence rationale. Therefore, we only required the model to predict one rationale sentence.

### CliVER: Baseline model

**Figure 1.**
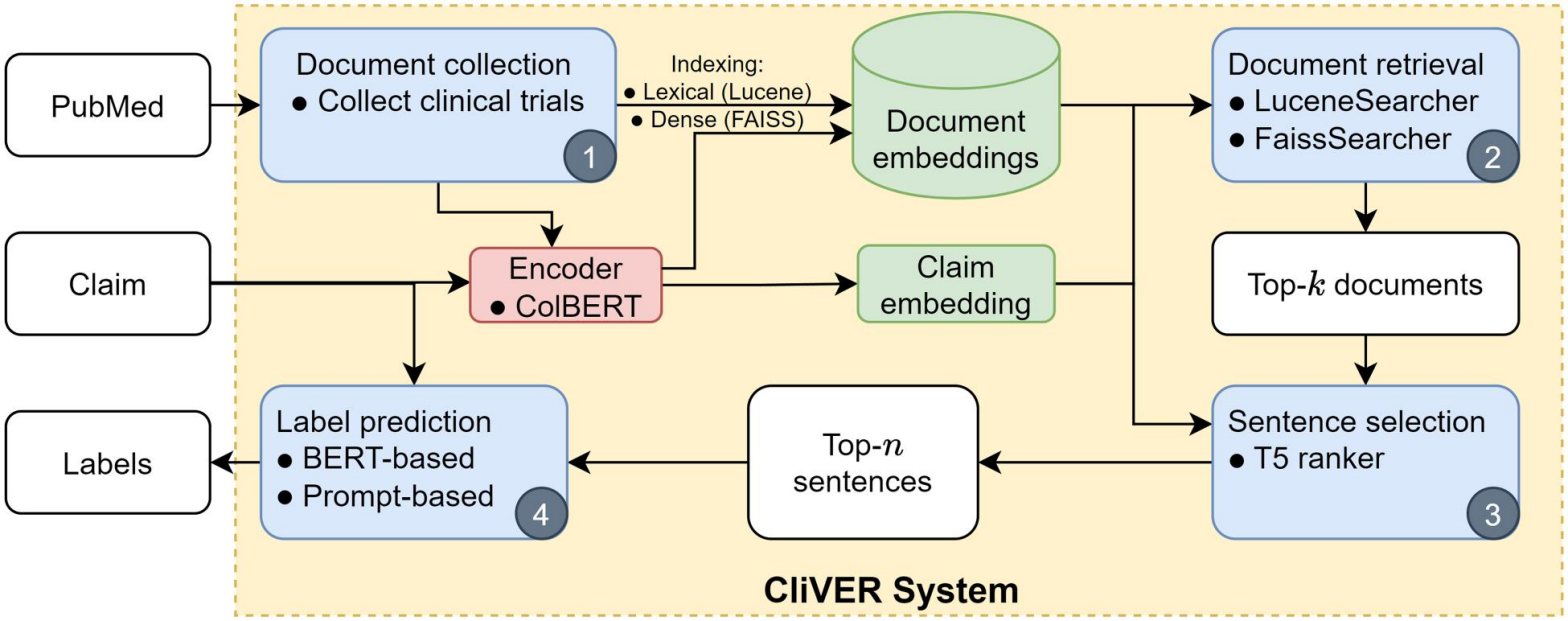
The overview of the CliVER framework with four major modules: Document collection, Document retrieval, Sentence selection, and Label prediction.

We developed CliVER with four modules (Figure 1).

#### Document collection

From PubMed API, we first identified PMIDs (PubMed unique ID) with a query parameter “term=(randomized+controlled+trial[all+fields])” (including all articles with “randomized controlled trial” in publication types or MeSH terms) and a time window from January 2010 to October 2021. Using the PMIDs, we downloaded a total of 189 648 clinical trial publications. For each publication, the title, abstract, and metadata (eg, authors, journal title, and publication date) were parsed and stored in JSON files. We created both lexical and dense indexes to enable efficient retrieval. Lexical indexes were built with Lucene to store term-to-document mappings for fast response to “bag of words” querying. Dense indexes were FAISS indexes built upon document-level vectors encoded with the pre-trained ColBERT model.^21^ Faiss supports efficient similarity search in sets of dense vectors.

#### Document retrieval

Given a scientific claim, this module retrieved the top-k clinical trial publications using a hybrid approach. A claim is encoded using the same ColBERT encoder for creating document embeddings. Then, we employed two similarity searchers, LuceneSearcher and FaissSearcher, to retrieve the n most relevant candidate articles to the claim. LuceneSearcher evaluates claim-article similarity using bag-of-words representations, while FaissSearcher uses dense transformer-derived representations. The articles identified by two searchers are ranked by the relevance score of a candidate article to the claim, with a pre-trained document rank model (T5 ranker^33^). The top-k articles were returned with respect to the estimated scores.

#### Sentence selection

Given the claim and each of the top-k articles, this module selects a top-t rationale sentence(s) from the abstract. For simplicity, we set t =1 to select one sentence with the highest similarity score as the primary rationale. To achieve this, we used the same document ranking model (T5 ranker) to rank similarities between the claim’s and each sentence’s embeddings.

#### Label prediction

Given the claim, the abstract, and their candidate rationale sentences, this module predicts the label of the abstract. We treated it as a multiclass classification problem, where the output indicates whether a given rationale sentence Supports, (is) Neutral, or Refutes a claim.

We hypothesized that an ensemble system combining the results of different methods could yield better predictive performance than a single method.^34^^,^^35^ Hence, we addressed the label prediction task using an ensemble system that combined the results from three individual models (Figure 1, module 4). Each individual system included two BERT-based models and one prompt-based model.

For the BERT-based approach, we concatenated a claim c and its corresponding sentence s with the special token [SEP] and added a prefix token [CLS] to form an input sequence [CLS, c, SEP, s], and fed this input to BERT. The model takes a sequence of tokens with a maximum length of 128 and produces a 768-dimensional sequence representation vector. For text shorter than 128 tokens, we added paddings (empty tokens) to the end of the text to make up the length. For text that is longer than 128 tokens, we used the first 128 tokens as the input. Then, a fully connected layer is appended on top of the [CLS] representation. Finally, a softmax layer maps the representation vector to the target label space. We trained the model with the objective function set to minimize cross-entropy loss between the predicted and manually annotated labels. We selected RoBERTa^36^ and PubMedBERT^37^ as our backbone models. RoBERTa was chosen because it demonstrated strong performance in previous work VERISCI.^12^ PubMedBERT was chosen because it was domain-specifically pre-trained on PubMed and obtained consistent gains in most BioNLP tasks.

The second is a prompt-based model. In this work, we used the prompt function $f_{\mathrm{prompt}}(c,s)=$ “MNLI Hypothesis: [c] Premise: [s] Target: [MASK]” to generate a prompt $x_{\mathrm{prompt}}$, which is a textual string that includes a one-token answer slot [MASK]. The language model then takes $x_{\mathrm{prompt}}$ as the input, maps it to a sequence of token embeddings, and learns to select one answer z to fill in the answer slot [MASK]. The highest-scored answer z is then mapped to l in the label space of {Support, Refute, Neural}. We trained a task-specific head by maximizing the log-probability of the correct label at the masked token, given the hidden vector of [MASK]. For the prompt-based approach, we selected T5 as our backbone model, because it demonstrated strong performance in the previous work.^13^

We leveraged the majority voting ensemble method to harmonize the results of the three models to obtain the final labels. Hence, the label with the highest number of votes from all three models will be selected. If all three votes are different, the vote from the T5 model will be picked as it was previously shown with better label prediction performance on SciFact.^12^

### Data for label prediction

To train and validate the Label Prediction model, we used four datasets. One is newly curated and the other three are publicly available (Table 1).

**Table 1.** Characteristics of the label prediction data.

				Claim sentence pairs		
Split	Dataset	Claims	Abstracts	Support	Refute	Neutral
Training	SciFact	809	565	332	173	304
	FEVER	6666	1328^a^	3333	1681	1652
	ManConCorpus	2694	259	1966	728	2000
	Total	10 169	2152	5631	2582	3956
Test	SciFact	300	283	124	64	112
	CoVERt	15	96	46	60	106
	Total	315	379	170	124	218

a Count of linked Wikipedia pages.

#### CoVERt (covid VERification dataset)

**Figure 2.**
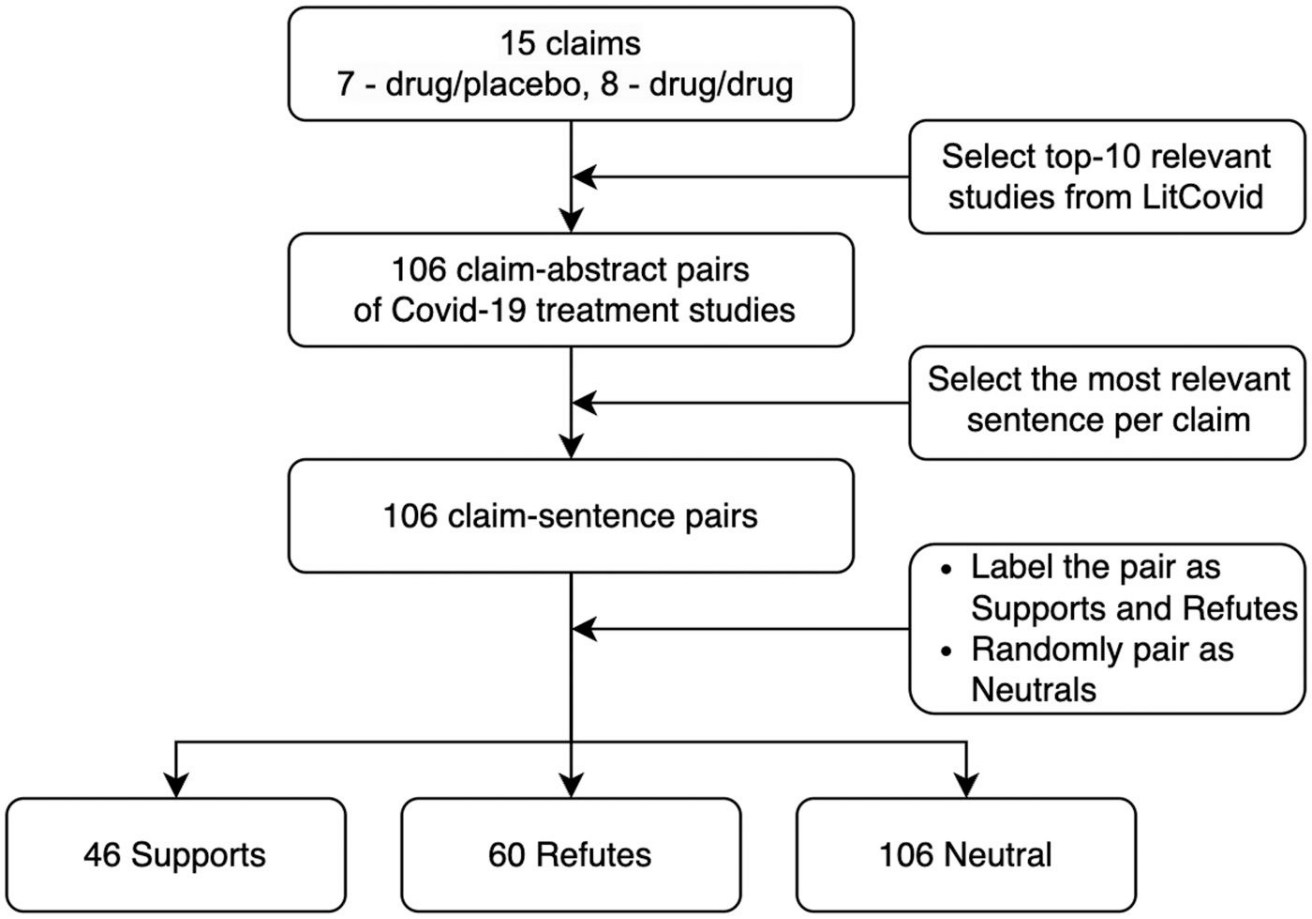
Flowchart of creation of CoVERt.

To assess the performance of the Label Prediction component in CliVER, we manually curated a new **Co**vid **VER**ification datase**t (CoVERt)** dataset (Figure 2). To generate CoVERt, we formulated 15 claims to evaluate 11 different drugs’ effects on treating COVID-19 to extract relevant studies on LitCovid, as shown in Supplementary Table S1. Eleven claims compared a drug to placebo/standard care and the remaining four claims were drug-to-drug comparisons.

We composed these claims to comply with the Population, Intervention, Comparison, and Outcome (PICO) elements.^38^ Empirical studies have shown that using the PICO search strategy improves formulating a good clinical research question.^32^ For example, given “Remdesivir” as the drug, “29-day mortality” as the outcome, and “COVID-19 patients” as the Population, we had the claim *“Remdesivir (I), compared to placebo/standard care (C), reduces the 29-day mortality (O) for treating COVID-19 patients (P).”*

For each claim, we first searched only treatment studies on LitCovid^2^ with target drug names and the location filter set to the United States for relevant abstracts. From the top-10 returned studies (latest first), a study was manually selected (JP and HL) if its’ abstract includes a conclusion about the drug effects or comparison for the queried claim. Later, the corresponding sentence that provides the most relevant evidence to the claim in each abstract was identified based on the consensus of two annotators (SL and MB) following the same annotation guide in ManConCorpus.^27^ A total of 96 abstracts were selected for 15 claims, and their most relevant sentences were extracted and used to form the sentence-claim pairs. Afterward, the annotators manually labeled pairs as *Support* or *Refute*, following the manual annotation procedure described by Alamri.^27^ Only labels with consensus (after discussion as needed) by both annotators were included. To provide *Neutral* class pairs, claims and non-relevant sentences were randomly paired to match the total number of cases in the *Support* and *Refute* classes. As a result, we had 212 sentence-claim pairs (ie, 46 *Support*, 106 *Neutral*, and 60 *Refute*). Table 2 lists examples.

**Table 2.** Examples of annotated paired claims and sentences in CoVERt.

Label	Claim	Corresponding sentence
Contradiction	Methylprednisolone (MP), compared to dexamethasone, improves clinical outcome for treating COVID-19 patients.	Dexamethasone and methylprednisolone both are equally effective in treating moderate to severe COVID-19 diseases. (PMID 33200031)
Support	Remdesivir, compared to placebo/standard care or none, improves clinical outcome for treating COVID-19 patients.	Our data show that Remdesivir was superior to placebo in shortening the time to recovery in adults who were hospitalized with Covid-19 and had evidence of lower respiratory tract infection. (PMID 32445440)
Neutral	Azithromycin, compared to placebo/standard care, improves clinical outcome for treating COVID-19 patients.	Remdesivir has shown in vitro activity against coronaviruses and is a possible antiviral treatment for SARS-CoV-2 infection. (PMID 32407959)

#### Other datasets

Besides CoVERt, we also used three publicly available corpora to train our label prediction module: SciFact, FEVER, and ManConCorpus (Table 1). Supplementary Table S2 provides examples of annotated claims and evidence sentences from SciFact, FEVER, and ManConCorpus.

**SciFact** is a collection of 1409 claims verified against 5183 abstracts. For each claim, the corresponding abstracts were annotated as Supports, Refutes, or Neutral (NoInfo) with rationale sentences (also referred to as “evidence”) from the abstracts. Since the ground truth for the test set was not published, we used the standard training set (809 claims) for training and the development set (600 claims) for testing.

**FEVER** (Fact Extraction and VERification) is a large general-domain fact-checking dataset consisting of 185 445 claims manually verified against the introductory sections of Wikipedia pages. The claims were further classified as Supported, Refuted, or NotEnoughInfo, with an inter-annotator agreement of 0.6841 in Fleiss kappa. In this study, NotEnoughInfo was mapped to Neutral. We used the version described in Lee et al. as part of the training set.^39^

**ManConCorpus** (MANually annotated CONtradiction Corpus) is another publicly available, manually annotated corpus. It contains 259 clinical trial abstracts, including 17 911 sentence pairs sorted into 24 groups. Each group answers a unique research question with labels being Contradiction (Refute), Entailment (Support), or Neutral. In this study, we used all contradiction (*N* = 728) and entailment (*N* = 1966) pairs and randomly sampled 2000 Neutral pairs for training.

### Evaluation design

**Figure 3.**
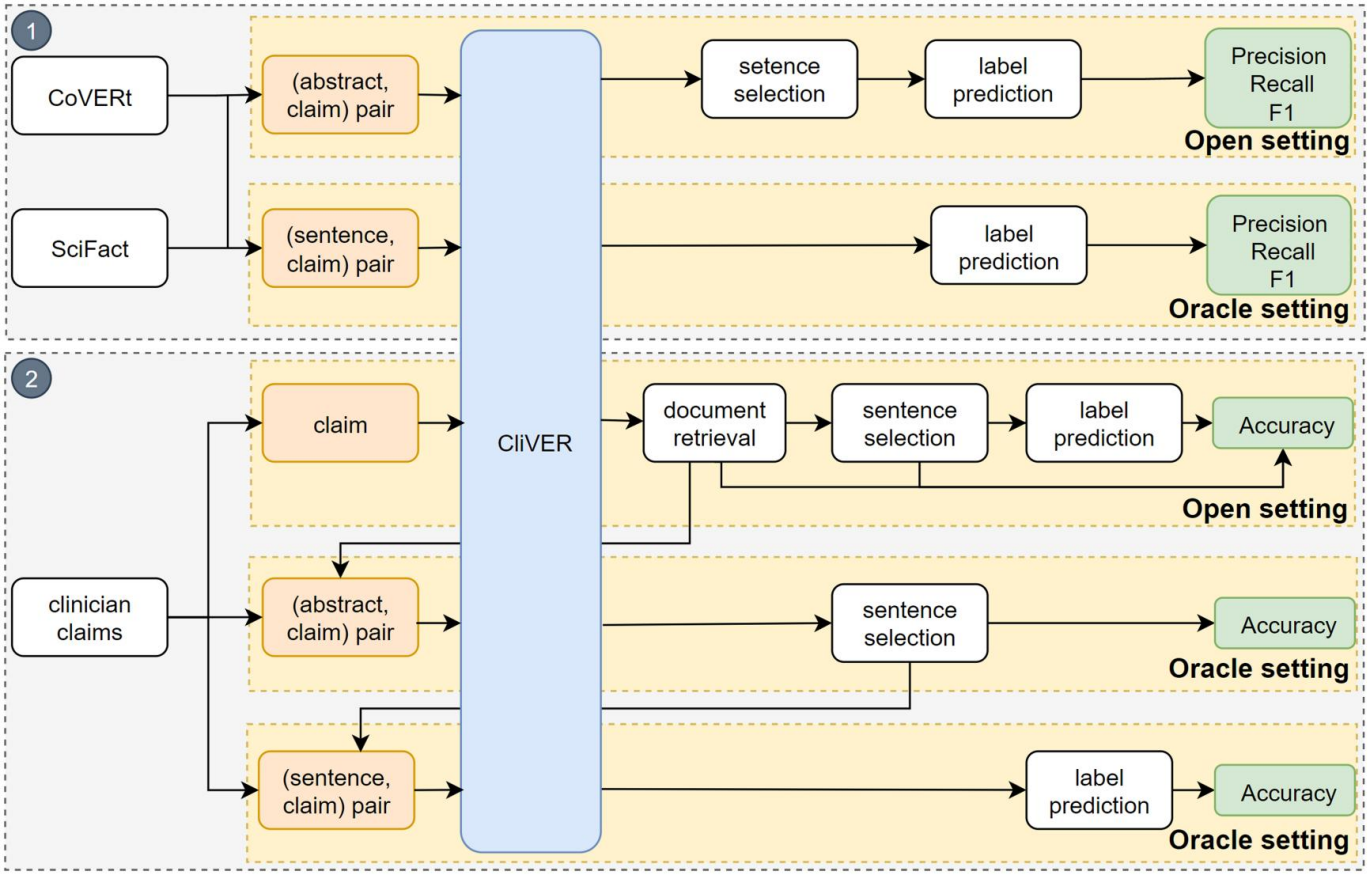
Overview of the two evaluations: (1) evaluation of CliVER’s label prediction module on SciFact and CoVERt datasets; (2) evaluation of CliVER’s agreement with human reviews in Open and Oracle setting on clinicians’ claims.

We designed two evaluations (shown in Figure 3):

1. We used two datasets to evaluate CliVER’s accuracy on label prediction (Figure 3(1)): SciFact’s development dataset and CoVERt. The precision, recall, and F1 scores were calculated.
2. We recruited clinicians to generate reference standards for evaluating CliVER’s overall performance in verifying real-world claims against human reviews (Figure 3(2)). We identified 19 clinical research claims from 6 disease categories. Results were compared with clinicians’ manual reviews to calculate accuracy, ie, the proportion of relevant studies extracted by CliVER, and the proportion of predicted labels agreed with the human reviewed outcome. We did not measure recall since we cannot exhaust reviewing all relevant abstracts in PubMed for each claim.

The results were reported in two settings: (1) in the Open setting, where only the claims are provided to CliVER, and (2) in the Oracle setting, where corresponding abstracts or sentences for a given claim are provided to CliVER. The Oracle setting simulates the ideal relevant abstract and sentence retrieval to assess label prediction performance. At the same time, the Open setting also assesses CliVER’s abstract and sentence selection performance in the context of a real-world use case scenario.

### Experimental settings

We first fine-tuned three individual models on the FEVER and ManConCorpus datasets for 3 epochs with a learning rate of 2 × 10^−5^. The batch size was 16. We adopted the AdamW optimizer^40^ as the optimizer. AdamW is a variant of the original Adam adaptive gradient algorithm^41^ using decoupled weight decay regularization. We then fine-tuned the models on the SciFact training dataset for 10 epochs with the AdamW optimizer, a learning rate of 1 × 10^−6^, and a batch size 16.

A random, stratified 15% of the training data was used as the development set. The whole framework was implemented using PyTorch, and all pre-trained base models were loaded from the HuggingFace^42^ platform, with the retrieval module (including sparse and dense indexing) implemented based on Pyserini.^43^ Intel Xeon^®^ Silver 4110 CPU 32 cores processor, NVIDIA RTX 2080Ti GPU, and a memory size of 128G were used in this work. We measured the performance by accuracy, precision, recall, F1 score, and macro-averaged F1 score.

## Results

### Performance of label prediction

We first evaluated the CliVER on label prediction in the Oracle setting. Table 3 shows the comparison of three individual models and their majority voting ensemble results on two testing datasets: SciFact’s development dataset and CoVERt. On SciFact, the Majority Voting ensemble achieved the best performance with a macro-average F1 score of 0.93. RoBERTa achieved the best individual model performance (0.89 in F1 score), followed by PubMedBERT (0.87) and T5 (0.79). However, PubMedBERT achieved the best Precision (0.95) for the Neutral class.

**Table 3.** Comparison of the three label prediction models and their majority voting ensemble results on SciFact’s development dataset and CoVERt, respectively.

Model	SciFact			CoVERt		
	Precision	Recall	F1 score	Precision	Recall	F1 score
RoBERTa (large)						
Support	0.90	0.91	0.90	0.70	**1.00**	0.82
Neutral	0.89	0.88	0.88	0.99	0.84	0.91
Refute	0.90	**0.91**	0.90	**0.93**	0.87	0.90
*Macro-average*			0.89			0.88
PubMedBERT						
Support	0.86	0.92	0.89	0.57	**1.00**	0.73
Neutral	**0.95**	0.85	0.90	**1.00**	0.73	0.84
Refute	0.82	0.85	0.83	0.89	0.82	0.85
*Macro-average*			0.87			0.81
T5 (base)						
Support	0.90	0.78	0.83	**0.97**	0.72	0.82
Neutral	0.77	0.90	0.83	0.90	**1.00**	**0.95**
Refute	0.73	0.72	0.72	0.90	0.90	0.90
*Macro-average*			0.79			0.89
Majority voting						
Support	**0.94**	**0.95**	**0.95**	0.79	**1.00**	**0.88**
Neutral	0.93	**0.95**	**0.94**	**1.00**	0.89	0.94
Refute	**0.93**	0.89	**0.91**	**0.93**	**0.93**	**0.93**
*Macro-average*			**0.93**			**0.92**

The precision, recall, and F1 score are reported, with the highest score for each metric highlighted in bold.

On the CoVERt dataset, the majority voting ensemble achieved the best overall performance with the highest macro-average F1 score (0.92). T5 obtained the best individual model performance, though there is no significant difference between T5 and RoBERTa (0.89 and 0.88). Interestingly, PubMedBERT received the best precision (1.0) for the Neutral class on both SciFact and CoVERt.

We further evaluated CliVER on whether it could identify sentences sufficient to justify the predictions in the Open setting. First, the sentence needs to be correctly selected and then it is correctly labeled. Supplementary Table S3 shows that the Majority Voting ensemble achieved the best performance with a macro-average F1 score of 0.85. As for the best individual model performance (in macro-average F1 score), RoBERTa (0.82) outperformed PubMedBERT (0.79) and T5 (0.79).

### Human evaluation of CliVER

Four medical clinicians (BI, AS, JN, and EP) with diverse clinical expertise were recruited. Each nominated five claims in their research domains. The topics cover Alzheimer’s disease, COVID-19, digestive diseases, hypertension, kidney diseases, and rheumatic diseases. One claim comparing the effectiveness of Adalimumab versus triple therapy (Methotrexate, Sulfasalazine, and Hydroxychloroquine) for rheumatic diseases was excluded because no qualifying study was found relevant to this claim in the extracted studies. As a result, we included 19 claims in the evaluation (as shown in Supplementary Table S4**)**.

#### Document retrieval

Given a claim, we considered the top-5 results returned by CliVER from a pool of indexed articles. Four evaluators used the 3-Likert scale to rate the relevance of abstracts in three points: (1) Relevant to claim, (2) Not relevant to claim, and (3) Non-RCT. In total, 95 abstracts were retrieved. Twelve non-RCT abstracts (4 protocol studies, 6 method-only studies, and 2 cost-effectiveness studies) were excluded due to the lack of outcome reports, eight abstracts were rated irrelevant to their pairing claims, and 75 were rated relevant, yielding an accuracy of 79.0%.

#### Sentence selection

We applied a 5-Likert scale survey on whether CliVER identified evidence sentences corresponding to the claim. Reviewers graded sentence relevance in five points: (1) Highly irrelevant; (2) Irrelevant; (3) Neither irrelevant nor relevant (Undecided); (4) Relevant, and (5) Highly relevant. We conducted evaluations in (1) the “Open” setting, where sentences from all abstracts returned by CliVER were included, and (2) the “Oracle” setting, where gold standard evidence sentences (rated by reviewers) were provided. The Open setting assesses the performance of sentence selection in real-world abstract retrieval, while the Oracle setting assesses the performance of sentence selection independently from other components.

Table 4 shows the manual review results for sentence selection. The complete evaluation results are provided in Supplementary Table S5. In the Oracle setting of sentence selection, for the 75 corresponding sentences (one in each abstract), evaluators determined that 59 were relevant (14 relevant, 45 highly relevant), yielding an accuracy of 78.7%. In the Open setting, for the 95 corresponding sentences, evaluators determined that 64 were relevant (18 relevant, 46 highly relevant), yielding an accuracy of 67.4%.

**Table 4.** Manual review results of CliVER’s performance.

Sentence selection			
Setting	5-Likert scale	Count	Accuracy (%)
**Oracle**			78.7
	Highly irrelevant	0	
	Irrelevant	5	
	Undecided	11	
	Relevant	14	
	Highly relevant	45	
**Open**			67.4
	Highly irrelevant	9	
	Irrelevant	6	
	Undecided	16	
	Relevant	18	
	Highly relevant	46	

Label prediction			
Setting	5-Likert scale	Count	Accuracy (%)
**Oracle**			79.7
	Strongly disagree	4	
	Somewhat disagree	3	
	Undecided	5	
	Agree	6	
	Strongly agree	41	
**Open**			63.2
	Strongly disagree	22	
	Somewhat disagree	5	
	Undecided	8	
	Agree	15	
	Strongly agree	45	

#### Label prediction

We applied a 5-Likert scale survey on whether CliVER correctly assigned labels (Support, Neutral, or Refute) to evidence sentences. Reviewers rated their agreement with the label in five points: (1) Strongly disagree; (2) Disagree; (3) Neither agree nor disagree (Undecided); (4) Agree; and (5) Strongly agree. We also conducted evaluations in two settings. The Open setting included all the sentences extracted by CliVER. The Oracle setting included gold sentences confirmed to be relevant to each claim (rated by reviewers in the Sentence selection).

Table 4 reports reviewers’ ratings for predicted labels in the Oracle setting (with the 59 sentences confirmed relevant by reviewers) and the Open setting (with all 95 extracted sentences). In the Oracle setting, reviewers agreed with 47 (6 agree, 41 strongly agree) machine-predicted labels, yielding an accuracy of 79.7%. In the Open setting, reviewers agreed with 60 (15 agree, 45 strongly agree) machine-predicted labels, and the accuracy was 63.2%.

#### Performance comparison across disease domains

**Figure 4.**
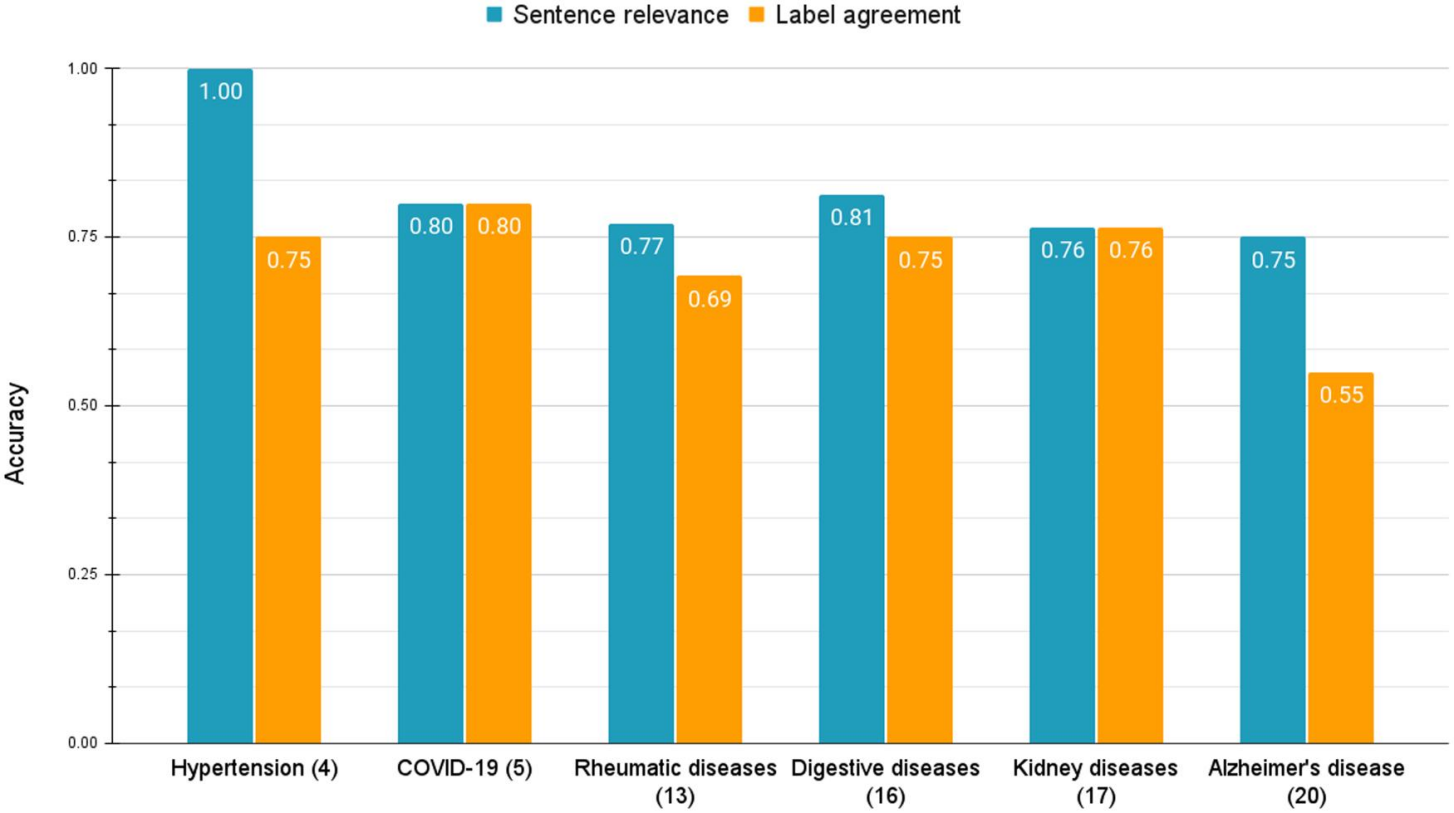
Accuracy of CliVER under the Oracle setting across six disease domains: Hypertension (4), COVID-19 (5), Rheumatic diseases (13), Digestive diseases (16), Kidney diseases (17), Alzheimer's disease (20). Study counts for each domain are shown in parentheses.

Figure 4 reports an Oracle setting analysis of the distribution of the 75 relevant abstracts in six disease domains: hypertension (4), COVID-19 (5), rheumatic diseases (13), digestive diseases (16), kidney diseases (17), and Alzheimer's disease (20). For each disease domain, we calculated the sentence-level relevance score (in blue) and the reviewer’s agreement with CliVER predicted labels (in red), under the Oracle setting of sentence selection. Except for hypertension, CliVER achieved similar sentence relevance scores (ranging from 0.75 to 0.81). Still, the label agreement accuracy varied, where the lowest accuracy (0.55) was for Alzheimer’s disease, and the highest accuracy (0.76) was for kidney diseases.

**Figure 5.**
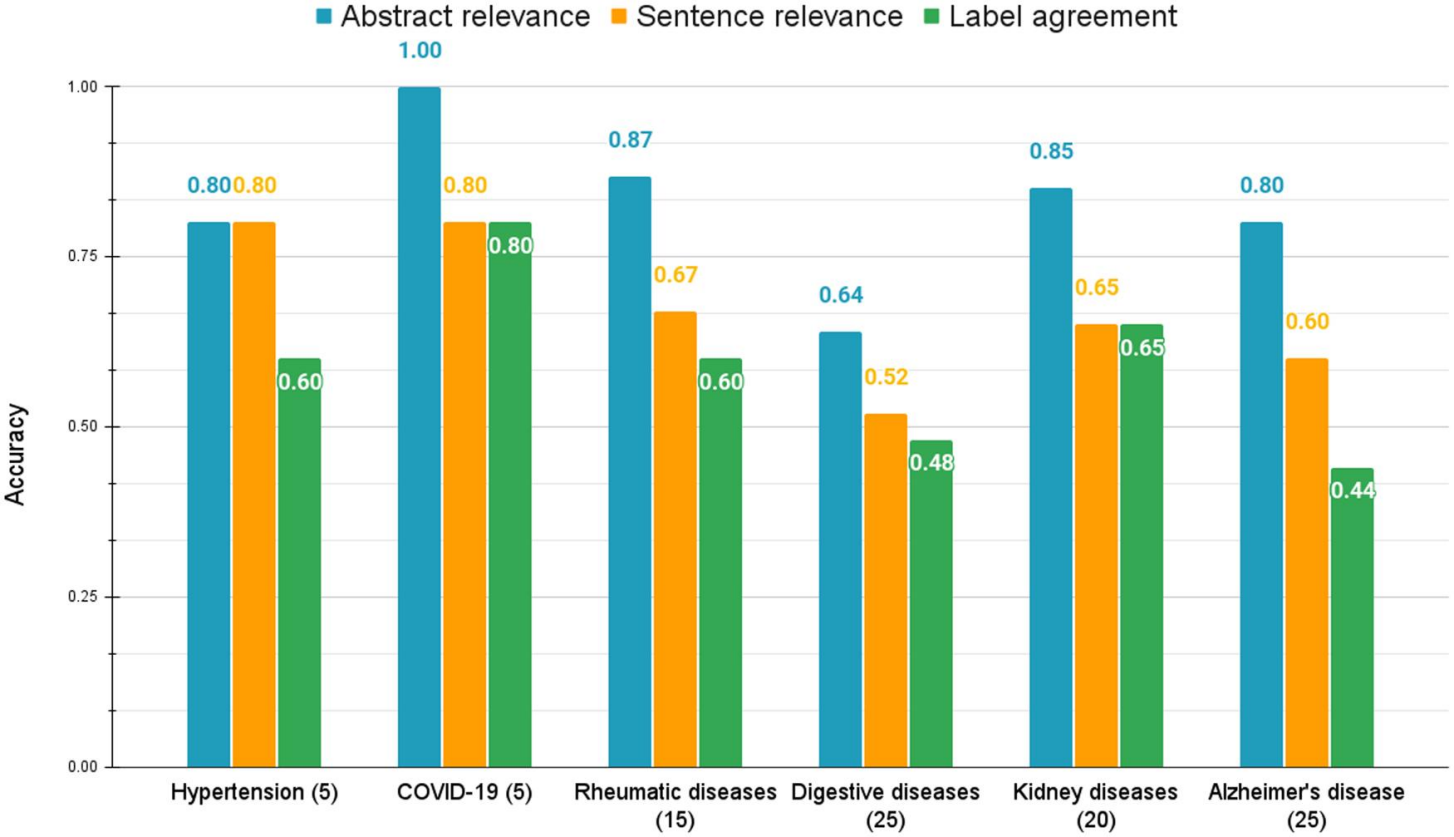
Accuracy of CliVER under Open setting across six disease domains: Hypertension (5), COVID-19 (5), Rheumatic diseases (15), Digestive diseases (25), Kidney diseases (20), Alzheimer's disease (25). Study counts for each domain are shown in parentheses.

Figure 5 reports an Open setting analysis of the distribution of the 95 relevant abstracts in six disease domains: hypertension (5), COVID-19 (5), rheumatic diseases (15), digestive diseases (25), kidney diseases (20), and Alzheimer's disease (20). For each disease domain, we calculated the abstract-level relevance score (in blue), sentence-level relevance score (in orange), and the reviewer’s agreement with CliVER predicted labels (in green), under the Open setting. Except for digestive diseases, CliVER achieved abstract-level relevance scores over 0.8 (ranging from 0.80 to 1.0). For sentence-level relevance retrieval, CliVER achieved similar scores (ranging from 0.52 to 0.67) in four domains with at least three claims (corresponding to more than 15 relevant studies). The label agreement accuracy varied, where the lowest accuracy (0.44) was for Alzheimer’s disease and the highest accuracy (0.80) was for the COVID-19 category.

In addition, we evaluated CliVER’s performance of ranking relevant abstracts with three rank-aware metrics, Precision@K, Mean Reciprocal Rank@K (MRR@K), and Normalized Discounted Cumulative Gain@K^44^^,^^45^ (NDCG@K). We evaluated the performance using 95 abstracts (with clinician-rated relevance) retrieved for 19 claims. With five (K = 5) abstracts ranked for each claim, we obtained an average Precision@5 score of 0.67, an MRR@5 of 0.89, and an averaged NDCG@5 score of 0.72 (full results in Supplementary Figure S6).

## Discussion

In the conventional clinical evidence appraisal process, clinicians or domain experts are tasked with the laborious manual search and selection of studies, and time-consuming discernment of relevant and high-quality findings. This conventional methodology poses inherent challenges, including inefficiency and the potential for oversight due to the vast volume of available literature. To address these limitations, we proposed to adopt the Retrieval Augmented approach that leverages embeddings for the indexing of research studies. This innovative methodology integrates vector-based semantic similarity search techniques, automatically identifying pertinent and high-quality findings from studies. By automating the identification of relevant and high-quality evidence, this approach has the potential of mitigating the burden on clinicians and domain experts and uncovering previously overlooked insights.

### New baseline of categorizing RCT studies for evidence appraisal

CliVER automatically retrieves clinical trial abstracts relevant to a scientific claim and classifies the abstracts as supporting or refuting evidence. It is a novel framework that adapted general scientific fact-checking models for automatic claim verification against hundreds of thousands of clinical research publications. CliVER shows early promise to efficiently and accurately identify relevant evidence for verifying a clinical scientific claim. The proposed system is the first of its kind to tackle the time and labor-intensive problem of appraising clinical evidence in the medical research literature.

Our ensemble model is an effective solution that harmonizes the output of different models, leveraging their complementary features to generate a single decision output. As shown in Table 3, no single model triumphs over all the others. Each of the three models has its advantage in specific scenarios over the others in label prediction. On SciFact’s development set, by applying the majority vote ensemble, we achieved the highest F1 score for each label prediction class and a 4% absolute increase in macro-average F1 score (0.93) compared to the individual model with the best performance (0.89). Similarly, when testing on CoVERt, the ensemble method outperformed the T5 (base) model (0.92 vs 0.89) by a 3% absolute increase in macro-average F1.

Another observation on evaluation with CoVERt using ensemble is that the *Support* class had a high recall of 1.0 but a low precision of 0.79, indicating that all the true *Support* cases were successfully predicted, but 21% of the cases predicted with the *Support* label were false *Support* cases. This may be because the imbalanced dataset (46 Support, 60 Refute, 106 Neutral) introduces statistical disparity among classes. For the Support class, the number of negative cases (60 Refute + 106 Neutral) was more than 3 times larger than the positive cases. Thus, even at a relatively low false positive rate, the False Positives could overwhelm the True Positives to yield a low precision.

For the *Neutral* class, we observed a higher precision of 1.0 and a lower recall of 0.89 on CoVERt compared to those on the SciFact dataset. This is probably because in SciFact, claims and evidence of “neutral” pairs cover a variety of disease domains (eg, claim: “*There is no association between HNF4A mutations and diabetes risks*,” evidence: “*Factors that predict macrosomia are poorly understood*.”). However, the claim and evidence pair in CoVERt deemed as “neutral” were still generally relevant because they were all within the scope of COVID-19 (annotated as Neutral in Table 1). Models trained from general neutral cases face the challenge of retrieving all neutral cases specific to a disease domain.

### Claim variation evaluation

We investigated CliVER’s performance towards different semantic variations of the input claims. Specifically, we generated four distinctive representations of PICO entities using templates listed below to mitigate the possible effects of template diversities. These templates are created with reference to an established evidence-based practice guideline.^46^ Additionally, we include a word (called Scale Indicator) to indicate the scale of the comparison frequently used in the comparative structures (eg, “improve”, “reduce”, “higher”, and “lower”).^47^^,^^48^ For each claim (formatted in Template 1) in CoVERt, we created three claims using Templates 2-4. Then, each generated claim was paired with the original claim’s rationale sentence for label prediction.

1. [Intervention], compared to [Comparator], [Scale Indicator] the [Outcome] for treating [Population].
2. In [Population], the effect of [Intervention], compared with [Comparator] is [Scale Indicator] on [Outcome].
3. For [Population], [Intervention] relative to [Comparator] [Scale Indicator] the risk for [Outcome].
4. For [Population], [Intervention] yields [Scale Indicator] than [Comparator] about [Outcome].

We reported the ensemble model’s label prediction results on CoVERt for four types of PICO templates (in Supplementary Table S7). We observed that the majority voting ensemble model achieved the best performance when claims were formatted using Template 2, with a macro-average F1 score of 0.93. The model had similar performance levels with Templates 1 and 3 (0.92). However, there was a slight drop in performance when using Template 4 (0.89). This suggests further studies are needed to develop a model with a more comprehensive set of PICO question templates in different clinical domains, such as intervention, diagnosis, etiology, prevention, prognosis, quality of life, and therapy.

### Error analysis for label prediction

We reviewed the narrative feedback from four clinicians and categorized the 12 label prediction errors into the following three types, all due to clinical knowledge incompleteness in the CliVER system (times of errors in parentheses).

**Error Type 1**: **Missing clinical context (5)**

**Example:** Claim 9— “*A restrictive transfusions strategy reduces death in patients with acute gastrointestinal bleeding.*”

**Corresponding sentence**: “*Restrictive transfusion strategy is non-inferior to liberal transfusion strategy in patients with UGIB*.”

**CliVER predicted label**: *Support*

**Clinician predicted label**: *Refute*

**Challenge:** It requires drawing conclusions using domain-specific contextual knowledge to determine if liberal transfusion strategy is the standard treatment for UGIB patients, especially when the comparator is implied but not specified in the claim.

**Error Type 2**: **Missing clinical significance scale (4)**

**Example:** For Claim 11—“*Cold snare polypectomy (CSP) is superior/non-inferior to hot snare polypectomy (HSP) for bleeding risk in patients undergoing colonoscopy.*”

**Corresponding sentence**: “*Intraprocedural bleeding was significantly more frequent in the CSP group than the HSP group (CSP, 19/208; HSP, 2/206; P<0.001) but resolved spontaneously without any intervention in both groups.*”

**CliVER predicted label**: *Support*

**Clinician predicted label**: *Refute*

**Challenge:** It requires an understanding of the clinical significance to compare variants of an outcome measure. For instance, intraprocedural bleeding is less clinically important than postpolypectomy bleeding.

**Error Type 3**: **Missing PICO alignment (3)**

**Example:** For Claim 16—“*Tolvaptan is associated with decreased total kidney volume in patients with Autosomal dominant polycystic kidney disease (ADPKD).*”

**Corresponding sentence**: “*Among subjects receiving tolvaptan, those with a greater suppression of Uosm had slower renal function decline.*”

**CliVER predicted label**: *Support*

**Clinician predicted label**: *Neutral*

**Challenge:** It requires refining our system’s extraction for better alignment with the desired intervention or outcome in a claim.

### Limitations and future work

In CliVER’s clinician evaluation, we sampled the claims from only a handful of disease domains. The major confounder of this evaluation is that claims from each disease domain were constructed by only one clinician. Therefore, our claim sampling strategy may neither represent the corresponding disease domain nor diverse clinicians in that domain. For instance, we observed that claims (nominated by a single clinician) for Alzheimer’s disease regarding under-investigated novel interventions with unspecific outcomes, such as “Lithium is safe” (claim 1) and “Cocoa improves memory” (claim 5), caused most label disagreements (Figures 4 and 5). In the future, we will test CliVER with more diverse claims, such as different semantics or syntaxes from a wide range of potential clinician users.

Multiple factors contribute to the imperfect performance of the current version of CliVER. RCT studies typically investigate binary outcomes of interventions, such as superiority, inferiority, non-inferiority, effective, and non-effective. This leaves out many observational or descriptive studies that also inform clinical guidelines (depending on the rarity or accessibility of diseases). With a future goal of assisting authors who need to perform meta-analyses, we plan to include observational studies in CliVER to support more comprehensive evidence extraction from clinical literature.

Another limitation is that abstract and sentence ranking modules were implemented with a T5 model fine-tuned with the MS MARCO dataset. While MS MARCO serves as an open-domain benchmark for machine reading comprehension and question answering, its suitability in biomedicine remains to be validated. In the future, we plan to incorporate a biomedical domain question-answering corpus to fine-tune the abstract ranking or sentence selection modules for improving evidence retrieval accuracy for scientific claim verification.

Finally, CliVER’s inference for veracity lacks interpretability, especially for lay people. The curation of CoVERt focused on detecting contradictions regarding treatment effectiveness, such as the contradiction between “*Remdesivir treatment was not associated with statistically significant clinical benefits*” and “*Remdesivir treatment was associated with decreased mortality rate”* and hence did not include data for other types of contradictions. For example, CliVER currently does not differentiate qualitative expressions or the impact of patient populations. For instance, abstracts showing small or large benefits will be labeled as “having benefits” regardless of patient population size. A potential approach to overcome this limitation is to leverage PICO entities in both the claim and the abstract. In addition, medical ontologies can be leveraged to standardize medical terms and expand synonyms in the retrieval process to identify implicit evidence. Translation of layman’s terms to formal terminology terms used in clinical studies, such as from “Pfizer covid-19 vaccine” to “BNT162b2 mRNA vaccine” (formally used in clinical trials), can potentially improve the usability of CliVER for laypeople. These approaches promise to narrow the scope of searching and improve the interpretability of verifying a claim’s veracity.

## Conclusion

We present CliVER, an automated end-to-end claim verification system that performs fact-checking against clinical trial literature from PubMed. Our experiment revealed that CliVER achieved accurate performance in Oracle and Open settings when validating claims from various health domains with clinicians’ manual reviews. The enhancement of retrieval-augmented methodologies in inferencing the relationship between scientific claims and scientific literature reveals the potential to elevate the precision and efficiency of claim verification. We also demonstrated that the ensemble model outperforms all its constituents (two transformer-based models and one sequence-to-sequence model) for inferencing the relationship between scientific claims and scientific literature. With the assistance of CliVER, researchers can quickly and accurately characterize the confidence in a particular clinical research claim. We hope CliVER can be leveraged to reduce the labor of collecting and screening studies towards evidence appraisal against vast clinical literature. It also has the potential to increase the productivity of clinical research by enabling researchers to keep pace with the rapid growth of literature and stay tuned with the latest findings.

## Supplementary Material

ooae021_Supplementary_Data

## Data Availability

The data underlying this article are available in the article and its online supplementary material.
