# Supplementary material for: Retrieval augmented scientific claim verification
Source: JAMIA Open. 2024 Feb 21;7(1):ooae021. doi: 10.1093/jamiaopen/ooae021 (PMC10919922; doi:10.1093/jamiaopen/ooae021)
Supplement: ooae021_Supplementary_Data [file ooae021_supplementary_data.docx]

**Supplements**

**The content of this supplements is:**

1. **Supplement Table S1.** 15 COVID-19 treatment related claims for 11 different drugs, with COVID-19 patients being the participants and clinical improvement being the primary outcomes.
2. **Supplement Table S2.** Examples of annotated claims and the corresponding sentences from SciFact, FEVER, and ManConCorpus datasets.
3. **Supplement Table S3.** Performance of the sentence selection + label prediction on Supports and Refutes claim-sentence pairs of CoVERt.
4. **Supplement Table S4.** 19 clinical research claims of six disease categories curated by four clinicians.
5. **Supplement Table S5.** The complete evaluation results of 19 claims nominated by four clinicians on 75 studies and corresponding sentences extracted by CliVER.
6. **Supplement Figure S6.** The Precision@5, RR@5, and NDCG@5 scores for each claim extracted by CliVER.
7. **Supplement Table S7.** Comparison of the label prediction results of four different PICO-templates for claims on CoVERt.

**Supplement Table S1**. 15 COVID-19 treatment related claims for 11 different drugs, with COVID-19 patients being the participants and clinical improvement being the primary outcomes.

| **No** | **Claim** | **Intervention** | **Comparator** |
| --- | --- | --- | --- |
| **1** | *Remdesivir, compared to placebo/standard care, improves clinical outcome for treating COVID-19 patients.* | Remdesivir | placebo/ standard care |
| **2** | *Remdesivir, compared to convalescent plasma, improves clinical outcome for treating COVID-19 patients.* | Remdesivir | convalescent plasma |
| **3** | *Chloroquine or hydroxychloroquine (HCQ), compared to placebo/standard care, improves clinical outcome for treating COVID-19 patients.* | Chloroquine or hydroxychloroquine | placebo/ standard care |
| **4** | *Chloroquine or hydroxychloroquine (HCQ), compared to azithromycin, improves clinical outcome for treating COVID-19 patients.* | Chloroquine or hydroxychloroquine | azithromycin |
| **5** | *Tocilizumab (TCZ), compared to placebo/standard care, improves clinical outcome for treating COVID-19 patients.* | Tocilizumab | placebo/ standard care |
| **6** | *Ivermectin, compared to placebo/standard care, improves clinical outcome for treating COVID-19 patients.* | Ivermectin | placebo/ standard care |
| **7** | *Azithromycin, compared to placebo/standard care, improves clinical outcome for treating COVID-19 patients.* | Azithromycin | placebo/ standard care |
| **8** | *Dexamethasone, compared to placebo/standard care, improves clinical outcome for treating COVID-19 patients.* | Dexamethasone | placebo/ standard care |
| **9** | *Sarilumab, compared to placebo/standard care, improves clinical outcome for treating COVID-19 patients.* | Sarilumab | placebo/ standard care |
| **10** | *Convalescent plasma (CCP), compared to placebo/standard care, improves clinical outcome for treating COVID-19 patients.* | Convalescent plasma | placebo/ standard care |
| **11** | *Methylprednisolone (MP), compared to placebo/standard care, improves clinical outcome for treating COVID-19 patients.* | Methylprednisolone | placebo/ standard care |
| **12** | *Methylprednisolone (MP), compared to dexamethasone, improves clinical outcome for treating COVID-19 patients.* | Methylprednisolone | dexamethasone |
| **13** | *Anakinra, compared to placebo/standard care, improves clinical outcome for treating COVID-19 patients.* | Anakinra | placebo/ standard care |
| **14** | *Intravenous immunoglobulin (IVIG), compared to placebo/standard care, improves clinical outcome for treating COVID-19 patients.* | Intravenous immunoglobulin | placebo/ standard care |
| **15** | *Dexamethasone, compared to methylprednisolone (MP), improves clinical outcome for treating COVID-19 patients.* | Dexamethasone | methylprednisolone |

**Supplement** **Table S2**. Examples of annotated claims and the corresponding sentences from SciFact, FEVER, and ManConCorpus datasets.

|  | **Label** | **Claim** | **Corresponding sentence** |
| --- | --- | --- | --- |
| **SciFact** | **Refutes** | *1 in 5 million in UK have abnormal PrP positivity.* | *RESULTS Of the 32,441 appendix samples 16 were positive for abnormal PrP, indicating an overall prevalence of 493 per million population (95% confidence interval 282 to 801 per million).* |
|  | **Supports** | *ART substantially reduces infectiveness of HIV-positive people.* | *Considering the actual scale-up of ART in South Africa, seven models estimated that current HIV incidence is 17% to 32% lower than it would have been in the absence of ART.* |
|  | **Noinfo** | *0-dimensional biomaterials lack inductive properties.* | *This review examines the use of nanotechnologies for stem cell tracking, differentiation, and transplantation.* |
| **FEVER** | **Refuted** | *Red Headed Stranger is a movie.* | *Red Headed Stranger is a 1975 album by American outlaw country singer Willie Nelson.* |
|  | **Supported** | *There is a movie called The Hunger Games.* | *The Hunger Games is a 2012 American dystopian science fiction adventure film directed by Gary Ross and based on the novel of the same name by Suzanne Collins.* |
|  | **NotEnoughInfo** | *John Wick: Chapter 2 was theatrically released in the Oregon.* | *The series began in 2014 with the release of John Wick, which was written by Derek Kolstad, produced by Basil Iwanyk, Stahelski, Leitch, Eva Longoria and Michael Witherill, and followed by the sequel John Wick: Chapter 2, which was released in 2017.* |
| **ManConCorpus** | **Contradiction** | *The present study provides evidence that T-786C polymorphism of the NOS3 gene is associated with CAD.* | *These findings suggest that the 894G>T, -786T>C and 4a/4b polymorphisms of the NOS3 were not associated with CAD in the studied subjects.* |
|  | **Entailment** | *The present study provides evidence that T-786C polymorphism of the NOS3 gene is associated with CAD.* | *The -786T > C was the polymorphism associated with severe CAD in this study.* |
|  | **Neutral** | *The present study provides evidence that T-786C polymorphism of the NOS3 gene is associated with CAD.* | These findings further demonstrated that immunogenetics might play a predominant pathogenetic role in partial DCM patients. |

**Supplement Table S3**. Performance of the sentence selection + label prediction on *Supports* and *Refutes* claim-sentence pairs of CoVERt.

|  |  | | CoVERt |  |
| --- | --- | --- | --- | --- |
|  | Precision | Recall | | F1 score |
| RoBERTa (large) |  |  | |  |
| Support | 0.84 | **0.78** | | 0.81 |
| Refute | **1.00** | 0.72 | | 0.83 |
| *Macro-average* |  |  | | 0.82 |
| PubMedBERT |  |  | |  |
| Support | 0.78 | **0.78** | | 0.78 |
| Refute | **1.00** | 0.67 | | 0.80 |
| *Macro-average* |  |  | | 0.79 |
| T5 (base) |  |  | |  |
| Support | **0.96** | 0.59 | | 0.73 |
| Refute | 0.90 | **0.77** | | 0.83 |
| *Macro-average* |  |  | | 0.79 |
| Majority voting |  |  | |  |
| Support | 0.90 | **0.78** | | **0.84** |
| Refute | **1.00** | **0.77** | | **0.87** |
| *Macro-average* |  |  | | **0.85** |

**Supplement Table S4.** 19 clinical research claims of six disease categories curated by four clinicians.

| **Disease category** | **Claim** | |
| --- | --- | --- |
| Alzheimer's disease | 1. | Lithium is safe for individuals with Alzheimer's disease and agitation. |
|  | 2. | β amyloid and tau in the CSF can be detected in preclinical Alzheimer's disease. |
|  | 3. | Namenda (or memantine) is beneficial for mild Alzheimer's disease. |
|  | 4. | Regular exercise helps prevent cognitive decline in individuals diagnosed with mild cognitive impairment. |
|  | 5. | Cocoa improves memory in Alzheimer's disease patients. |
| COVID-19 | 6. | Tocilizumab (TCZ) improves clinical outcomes compared to standard care in patients admitted to hospital with COVID-19. |
| Digestive diseases | 7. | Screening colonoscopy reduces colon cancer related deaths in patients over 50. |
|  | 8. | Radiofrequency ablation reduces progression of disease in patients with Barrett's esophagus with dysplasia. |
|  | 9. | A restrictive transfusion strategy reduces death in patients with acute gastrointestinal bleeding. |
|  | 10. | Proton pump inhibitors (PPIs) are superior/non-inferior to histamine-2 (H2) receptor blockers for all-cause mortality in ICU patients. |
|  | 11. | Cold snare polypectomy (CSP) is superior/non-inferior to hot snare polypectomy (HSP) for bleeding risk in patients undergoing colonoscopy. |
| Hypertension | 12. | Intensive blood pressure control is associated with higher rates of all-cause mortality in nondiabetic patients with high blood pressure compared to standard blood pressure control. |
| Kidney diseases | 13. | Sodium zirconium is associated with a decrease in mean serum potassium levels among patients with hyperkalemia. |
|  | 14. | Intensive blood pressure control is associated with a greater decrease in total kidney volume than standard blood pressure control among hypertensive patients with Autosomal dominant polycystic kidney disease (ADPKD). |
|  | 15. | Lisinopril with telmisartan, compared to lisinopril alone, is associated with decreased rates of End-Stage Renal Disease (ESRD) among patients with Autosomal dominant polycystic kidney disease (ADPKD). |
|  | 16. | Tolvaptan is associated with decreased total kidney volume in patients with Autosomal dominant polycystic kidney disease (ADPKD). |
| Rheumatic diseases | 17. | Belimumab is superior/non-inferior to standard treatment for inducing renal remission in patients diagnosed with lupus nephritis. |
|  | 18. | Tofacitinib is superior/non-inferior to adalimumab for inducing clinical remission in patients diagnosed with rheumatoid arthritis. |
|  | 19. | Rituximab is superior/non-inferior to cyclophosphamide for inducing clinical remission in patients diagnosed with Antineutrophil cytoplasmic antibody (ANCA) vasculitis. |

**Supplement Table S5.** The complete evaluation results of 19 claims nominated by four clinicians on 75 studies and corresponding sentences extracted by CliVER.

| **Disease category** | **query** | **doc_id** | **sentence** | **machine predicted label** | **manual label** | **Is the extracted sentence relevant to the query? (On the scale of 1 to 5, with 1 as highly irrelevant, 2 as somewhat irrelevant, 3 as neutral, 4 as somewhat relevant, 5 as highly relevant.)** | **Do you agree with the assigned label for the extracted sentence, in terms of its relationship to the query? (On the scale of 1 to 5, with 1 as strongly disagree, 2 as somewhat disagree, 3 as neutral, 4 as somewhat agree, 5 as strongly agree.)** |
| --- | --- | --- | --- | --- | --- | --- | --- |
| Covid-19 | Tocilizumab (TCZ) improves COVID19 outcomes compared to standard care in patients admitted to hospital with COVID-19. | PMID33080017 | At day 14, 12% (95% CI -28% to 4%) fewer patients needed noninvasive ventilation (NIV) or mechanical ventilation (MV) or died in the TCZ group than in the UC group (24% vs 36%, median posterior hazard ratio [HR] 0.58; 90% CrI, 0.33-1.00), with a posterior probability of HR less than 1 of 95.0%, achieving the predefined efficacy threshold. | support | support | 5 | 5 |
| Covid-19 | Tocilizumab (TCZ) improves COVID19 outcomes compared to standard care in patients admitted to hospital with COVID-19. | PMID33676589 | However, post-hoc evidence from this study suggests tocilizumab might still be effective in patients with severe COVID-19 and so should be investigated further in future studies. | support | support | 5 | 5 |
| Covid-19 | Tocilizumab (TCZ) improves COVID19 outcomes compared to standard care in patients admitted to hospital with COVID-19. | PMID33080005 | In this randomized clinical trial of hospitalized adult patients with COVID-19 pneumonia and Pao2/Fio2 ratio between 200 and 300 mm Hg who received tocilizumab, no benefit on disease progression was observed compared with standard care. | contradiction | contradiction | 5 | 5 |
| Covid-19 | Tocilizumab (TCZ) improves COVID19 outcomes compared to standard care in patients admitted to hospital with COVID-19. | PMID33472855 | In patients with severe or critical covid-19, tocilizumab plus standard care was not superior to standard care alone in improving clinical outcomes at 15 days, and it might increase mortality. | support | contradiction | 5 | 1 |
| Covid-19 | Tocilizumab (TCZ) improves COVID19 outcomes compared to standard care in patients admitted to hospital with COVID-19. | PMID33831046 | There was no evidence to support an improvement in hypoxemia or ventilator-free survival with use of tocilizumab 400 mg in the absence of corticosteroids. | contradiction | contradiction | 3 | 5 |
| Rheumatic Diseases | Tofacitinib is superior/non-inferior to adalimumab for inducing clinical remission in patients diagnosed with rheumatoid arthritis. | PMID22873531 | In patients with rheumatoid arthritis receiving background methotrexate, tofacitinib was significantly superior to placebo and was numerically similar to adalimumab in efficacy. ( | support | support | 5 | 5 |
| Rheumatic Diseases | Tofacitinib is superior/non-inferior to adalimumab for inducing clinical remission in patients diagnosed with rheumatoid arthritis. | PMID21952978 | Treatment with tofacitinib at a dose of ÈàÆÔøΩ3 mg twice a day resulted in a rapid response with significant efficacy when compared to placebo, as indicated by the primary end point (ACR20 response at week 12), achieved in 39.2% (3 mg; P ÈàÆÔøΩ 0.05), 59.2% (5 mg; P < 0.0001), 70.5% (10 mg; P < 0.0001), and 71.9% (15 mg; P < 0.0001) in the tofacitinib group and 35.9% of patients in the adalimumab group (P = 0.105), compared with 22.0% of patients receiving placebo. | support | neutral | 3 | 1 |
| Rheumatic Diseases | Tofacitinib is superior/non-inferior to adalimumab for inducing clinical remission in patients diagnosed with rheumatoid arthritis. | PMID23515142 | Tocilizumab monotherapy was superior to adalimumab monotherapy for reduction of signs and symptoms of rheumatoid arthritis in patients for whom methotrexate was deemed inappropriate. | support | support | 5 | 5 |
| Rheumatic Diseases | Tofacitinib is superior/non-inferior to adalimumab for inducing clinical remission in patients diagnosed with rheumatoid arthritis. | PMID29045212 | The efficacy of tofacitinib was superior to that of placebo at month 3 in patients with psoriatic arthritis who had previously had an inadequate response to conventional synthetic DMARDs. | support | neutral | 3 | 1 |
| Rheumatic Diseases | Tofacitinib is superior/non-inferior to adalimumab for inducing clinical remission in patients diagnosed with rheumatoid arthritis. | PMID26929445 | Patients with moderate to severe RA and inadequate responses to MTX reported improvements across a broad range of PROs with tofacitinib 5 and 10 mg BID and adalimumab that were significantly superior to placebo. | support | neutral | 3 | 1 |
| Rheumatic Diseases | Rituximab is superior/non-inferior to cyclophosphamide for inducing clinical remission in patients diagnosed with Antineutrophil cytoplasmic antibody (ANCA) vasculitis. | PMID20647199 | CONCLUSIONS : Rituximab therapy was not inferior to daily cyclophosphamide treatment for induction of remission in severe ANCA-associated vasculitis and may be superior in relapsing disease. ( | support | support | 5 | 5 |
| Rheumatic Diseases | Rituximab is superior/non-inferior to cyclophosphamide for inducing clinical remission in patients diagnosed with Antineutrophil cytoplasmic antibody (ANCA) vasculitis. | PMID25381429 | In conclusion, patients with AAV and renal involvement respond similarly to remission induction with RTX plus glucocorticoids or CYC plus glucocorticoids. | support | support | 5 | 5 |
| Rheumatic Diseases | Rituximab is superior/non-inferior to cyclophosphamide for inducing clinical remission in patients diagnosed with Antineutrophil cytoplasmic antibody (ANCA) vasculitis. | PMID20647198 | CONCLUSIONS : A rituximab-based regimen was not superior to standard intravenous cyclophosphamide for severe ANCA-associated vasculitis. | support | contradiction | 5 | 1 |
| Rheumatic Diseases | Rituximab is superior/non-inferior to cyclophosphamide for inducing clinical remission in patients diagnosed with Antineutrophil cytoplasmic antibody (ANCA) vasculitis. | PMID23902481 | In patients with severe ANCA-associated vasculitis, a single course of rituximab was as effective as continuous conventional immunosuppressive therapy for the induction and maintenance of remissions over the course of 18 months. ( | support | support | 4 | 4 |
| Rheumatic Diseases | Rituximab is superior/non-inferior to cyclophosphamide for inducing clinical remission in patients diagnosed with Antineutrophil cytoplasmic antibody (ANCA) vasculitis. | PMID26621483 | CONCLUSIONS : Patients with PR3-AAV respond better to RTX than to CYC/AZA. | support | support | 5 | 5 |
| Kidney Diseases | Belimumab is superior/non-inferior to standard treatment for inducing renal remission in patients diagnosed with lupus nephritis. | PMID23263865 | Over 52 weeks, rates of renal flare, renal remission, renal organ disease improvement (assessed by Safety of Estrogens in Lupus Erythematosus National Assessment-Systemic Lupus Erythematosus Disease Activity Index and British Isles Lupus Assessment Group), proteinuria reduction, grade 3/4 proteinuria, and serologic activity favored belimumab, although the between-group differences in most renal outcomes were not significant. | support | support | 5 | 5 |
| Kidney Diseases | Belimumab is superior/non-inferior to standard treatment for inducing renal remission in patients diagnosed with lupus nephritis. | PMID32937045 | In this trial involving patients with active lupus nephritis, more patients who received belimumab plus standard therapy had a primary efficacy renal response than those who received standard therapy alone. ( | support | support | 5 | 5 |
| Kidney Diseases | Belimumab is superior/non-inferior to standard treatment for inducing renal remission in patients diagnosed with lupus nephritis. | PMID32755035 | Clinical efficacy was not improved with rituximab and CYC in combination with belimumab when compared to a therapeutic strategy of B cell depletion alone in patients with LN. | contradiction | contradiction | 4 | 4 |
| Alzheimer's disease | Lithium is safe for individuals with Alzheimer's disease and agitation. | PMID22746245 | As lithium is highly toxic in regular doses, our group evaluated the effect of a microdose of 300 μg, administered once daily on AD patients for 15 months. | support | neutral | 2 | 1 |
| Alzheimer's disease | Lithium is safe for individuals with Alzheimer's disease and agitation. | PMID21525519 | The present data support the notion that lithium has disease-modifying properties with potential clinical implications in the prevention of Alzheimer's disease. | support | neutral | 2 | 1 |
| Alzheimer's disease | Lithium is safe for individuals with Alzheimer's disease and agitation. | PMID21875410 | The findings of the present investigation indicated that beneficial effects of the lithium treatment might reduce the necessity of enhanced GDNF expression in the CNS in early AD. | support | neutral | 2 | 1 |
| Alzheimer's disease | β amyloid and tau in the CSF can be detected in preclinical Alzheimer's disease. | PMID29164798 | Individuals with a reduction in CSF Aβ levels (an indicator of amyloid accretion into neuritic plaques) as well as evident tau pathology (believed to be linked to neurodegeneration) exhibited lower subiculum volume, lower fornix microstructural integrity, and a trend towards lower cognitive score than individuals who showed only reduction in CSF Aβ. | support | neutral | 4 | 3 |
| Alzheimer's disease | β amyloid and tau in the CSF can be detected in preclinical Alzheimer's disease. | PMID27662296 | The reduction in CSF Aβ42, indicating β-amyloidosis, and increase in T-tau, indicating neurodegeneration, in hip fracture patients without dementia developing delirium indicates that preclinical AD brain pathology is clinically relevant and possibly plays a role in delirium pathophysiology. | support | support | 5 | 5 |
| Alzheimer's disease | β amyloid and tau in the CSF can be detected in preclinical Alzheimer's disease. | PMID22936010 | Optimal discrimination of AD and non-AD patients was achieved by combining Aβ(1-42) and P-tau(181P) (diagnostic accuracy = 0.86). | neutral | neutral | 4 | 4 |
| Alzheimer's disease | β amyloid and tau in the CSF can be detected in preclinical Alzheimer's disease. | PMID22531418 | Our data shows that CSF levels of Aβ(1-15/16) increase during treatment with semagacestat supporting its feasibility as a pharmacodynamic biomarker for drug candidates aimed at inhibiting Á∫¨-secretase-mediated AβPP-processing. | neutral | neutral | 3 | 3 |
| Alzheimer's disease | β amyloid and tau in the CSF can be detected in preclinical Alzheimer's disease. | PMID23438677 | The levels of Aβ (Aβ40 and Aβ42) and total tau in the CSF were determined. | support | neutral | 2 | 2 |
| Alzheimer's disease | Namenda (or memantine) is beneficial for mild Alzheimer's disease. | PMID21971472 | A double-blind placebo-controlled study was designed to measure the effect of a year-long course of memantine in patients with a probable AD diagnosis with mild to moderate dementia. | neutral | neutral | 5 | 5 |
| Alzheimer's disease | Namenda (or memantine) is beneficial for mild Alzheimer's disease. | PMID22397651 | Patients assigned to receive memantine, as compared with those assigned to receive memantine placebo, had a score on the SMMSE that was an average of 1.2 points higher (95% CI, 0.6 to 1.8; P<0.001) and a score on the BADLS that was 1.5 points lower (95% CI, 0.3 to 2.8; P=0.02). | support | support | 5 | 5 |
| Alzheimer's disease | Namenda (or memantine) is beneficial for mild Alzheimer's disease. | PMID24381967 | In the memantine group, these scores declined 1.98 units less (95% CI, -0.24 to 4.20; adjusted P = .40) than the placebo group's decline. | support | support | 5 | 5 |
| Alzheimer's disease | Namenda (or memantine) is beneficial for mild Alzheimer's disease. | PMID22567095 | Memantine was significantly better than placebo for cognition. | support | support | 5 | 5 |
| Alzheimer's disease | Regular exercise helps prevent cognitive decline in individuals diagnosed with mild cognitive impairment. | PMID31868666 | Greater cognitive benefits were achieved in the choreographic intervention than in the multimodal physical therapy, mainly in those functions more related to the risk of conversion to dementia. | support | support | 5 | 5 |
| Alzheimer's disease | Regular exercise helps prevent cognitive decline in individuals diagnosed with mild cognitive impairment. | PMID23390362 | General cognitive function was associated with improvements in physical performance after exercise intervention in subjects with mild cognitive impairment. | support | support | 5 | 5 |
| Alzheimer's disease | Regular exercise helps prevent cognitive decline in individuals diagnosed with mild cognitive impairment. | PMID29153754 | Combined physical and cognitive activity improves or maintains cognitive and physical performance in older adults with mild cognitive impairment, especially the amnestic type. | support | support | 5 | 5 |
| Alzheimer's disease | Regular exercise helps prevent cognitive decline in individuals diagnosed with mild cognitive impairment. | PMID23585901 | The results suggested that an exercise intervention is beneficial for improving logical memory and maintaining general cognitive function and reducing whole brain cortical atrophy in older adults with amnestic MCI. | support | support | 5 | 5 |
| Alzheimer's disease | Cocoa improves memory in Alzheimer's disease patients. | PMID25733639 | This dietary intervention study provides evidence that regular CF consumption can reduce some measures of age-related cognitive dysfunction, possibly through an improvement in insulin sensitivity. | neutral | neutral | 5 | 3 |
| Alzheimer's disease | Cocoa improves memory in Alzheimer's disease patients. | PMID22892813 | To the best of our knowledge, this is the first dietary intervention study demonstrating that the regular consumption of cocoa flavanols might be effective in improving cognitive function in elderly subjects with mild cognitive impairment. | support | support | 5 | 5 |
| Alzheimer's disease | Cocoa improves memory in Alzheimer's disease patients. | PMID23925758 | There is a strong correlation between neurovascular coupling and cognitive function, and both can be improved by regular cocoa consumption in individuals with baseline impairments. | neutral | support | 5 | 2 |
| Alzheimer's disease | Cocoa improves memory in Alzheimer's disease patients. | PMID22120044 | In the absence of significant behavioral effects, these differences in brain activation can be interpreted as evidence of increased neural efficiency in spatial working memory function associated with chronic cocoa flavanol consumption. | neutral | support | 5 | 2 |
| Digestive System Diseases | Screening colonoscopy reduces colon cancer related deaths in patients over 50. | [PMID24047060](https://pubmed.ncbi.nlm.nih.gov/24047060) | Screening reduced colorectal-cancer mortality (relative risk with annual screening, 0.68; 95% confidence interval [CI], 0.56 to 0.82; relative risk with biennial screening, 0.78; 95% CI, 0.65 to 0.93) through 30 years of follow-up. | support | support | 5 | 5 |
| Digestive System Diseases | Screening colonoscopy reduces colon cancer related deaths in patients over 50. | [PMID22612596](https://pubmed.ncbi.nlm.nih.gov/22612596) | Screening with flexible sigmoidoscopy was associated with a significant decrease in colorectal-cancer incidence (in both the distal and proximal colon) and mortality (distal colon only). ( | support | support | 5 | 5 |
| Digestive System Diseases | Radiofrequency ablation reduces progression of disease in patients with Barrett's esophagus. | [PMID24668102](https://pubmed.ncbi.nlm.nih.gov/24668102) | In this randomized trial of patients with Barrett esophagus and a confirmed diagnosis of low-grade dysplasia, radiofrequency ablation resulted in a reduced risk of neoplastic progression over 3 years of follow-up. | support | support | 5 | 5 |
| Digestive System Diseases | Radiofrequency ablation reduces progression of disease in patients with Barrett's esophagus. | [PMID21679712](https://pubmed.ncbi.nlm.nih.gov/21679712) | In subjects with dysplastic BE, RFA therapy has an acceptable safety profile, is durable, and is associated with a low rate of disease progression, for up to 3 years. | support | support | 5 | 5 |
| Digestive System Diseases | Radiofrequency ablation reduces progression of disease in patients with Barrett's esophagus. | [PMID30076843](https://pubmed.ncbi.nlm.nih.gov/30076843) | Adverse events (including stricture rate after starting RFA 3/36 [8.3%] and APC 3/37 [8.1%]) and quality of life scores were similar, but RFA cost $27491 more per case than APC. | neutral | neutral | 2 | 5 |
| Digestive System Diseases | Radiofrequency ablation reduces progression of disease in patients with Barrett's esophagus. | [PMID21839994](https://pubmed.ncbi.nlm.nih.gov/21839994) | At 3 months after 1 RFA session, 86% of patients (25/29) had a CR. | support | support | 5 | 5 |
| Digestive System Diseases | A restrictive transfusion strategy reduces death in patients with acute gastrointestinal bleeding. | [PMID23281973](https://pubmed.ncbi.nlm.nih.gov/23281973) | As compared with a liberal transfusion strategy, a restrictive strategy significantly improved outcomes in patients with acute upper gastrointestinal bleeding. ( | support | support | 4 | 4 |
| Digestive System Diseases | A restrictive transfusion strategy reduces death in patients with acute gastrointestinal bleeding. | [PMID24063362](https://pubmed.ncbi.nlm.nih.gov/24063362) | Compared with a liberal transfusion strategy, a restrictive strategy significantly improved outcomes in patients with acute UGIB. | support | support | 4 | 4 |
| Digestive System Diseases | A restrictive transfusion strategy reduces death in patients with acute gastrointestinal bleeding. | [PMID32801255](https://pubmed.ncbi.nlm.nih.gov/32801255) | Restrictive transfusion strategy is non-inferior to liberal transfusion strategy in patients with UGIB. | support | contradiction | 5 | 1 |
| Digestive System Diseases | A restrictive transfusion strategy reduces death in patients with acute gastrointestinal bleeding. | [PMID25956718](https://pubmed.ncbi.nlm.nih.gov/25956718) | A large cluster randomised trial to assess the effectiveness of transfusion strategies for acute upper gastrointestinal bleeding is both feasible and essential before clinical practice guidelines change to recommend restrictive transfusion for all patients with acute upper gastrointestinal bleeding. | support | support | 3 | 1 |
| Digestive System Diseases | Proton pump inhibitors (PPIs) are superior/non-inferior to histamine-2 (H2) receptor blockers for all-cause mortality in ICU patients. | [PMID31950977](https://pubmed.ncbi.nlm.nih.gov/31950977) | Among ICU patients requiring mechanical ventilation, a strategy of stress ulcer prophylaxis with use of proton pump inhibitors vs histamine-2 receptor blockers resulted in hospital mortality rates of 18.3% vs 17.5%, respectively, a difference that did not reach the significance threshold. | support | support | 5 | 5 |
| Digestive System Diseases | Cold snare polypectomy (CSP) is superior/non-inferior to hot snare polypectomy (HSP) for bleeding risk in patients undergoing colonoscopy. | [PMID34115035](https://pubmed.ncbi.nlm.nih.gov/34115035) | The rate of adverse events was 0.6% (1/175) for CSP and 0% (0/157) for HSP, which showed the non-inferiority of CSP. | support | support | 4 | 5 |
| Digestive System Diseases | Cold snare polypectomy (CSP) is superior/non-inferior to hot snare polypectomy (HSP) for bleeding risk in patients undergoing colonoscopy. | [PMID21689363](https://pubmed.ncbi.nlm.nih.gov/21689363) | Intraprocedural bleeding was significantly more frequent in the CSP group than the HSP group (CSP, 19/208; HSP, 2/206; P<0.001) but resolved spontaneously without any intervention in both groups. | support | contradiction | 4 | 2 |
| Digestive System Diseases | Cold snare polypectomy (CSP) is superior/non-inferior to hot snare polypectomy (HSP) for bleeding risk in patients undergoing colonoscopy. | [PMID28970290](https://pubmed.ncbi.nlm.nih.gov/28970290) | The non-inferiority of CSP for complete resection compared with HSP was confirmed by the +0.8% (90% CI -1.0 to 2.7) complete resection rate (non-inferiority p<0.0001). | support | support | 3 | 5 |
| Digestive System Diseases | Cold snare polypectomy (CSP) is superior/non-inferior to hot snare polypectomy (HSP) for bleeding risk in patients undergoing colonoscopy. | [PMID34432770](https://pubmed.ncbi.nlm.nih.gov/34432770) | CONCLUSIONS Our study findings showed that at 1-week follow-up, cold polypectomy resulted in improved colonic mucosal healing, with a smaller ulcer diameter and fewer blood vessels, when compared with hot polypectomy. | support | support | 4 | 5 |
| Digestive System Diseases | Cold snare polypectomy (CSP) is superior/non-inferior to hot snare polypectomy (HSP) for bleeding risk in patients undergoing colonoscopy. | [PMID28898922](https://pubmed.ncbi.nlm.nih.gov/28898922) | No clinically significant postprocedural bleeding or perforation occurred in either group. | contradiction | support | 4 | 1 |
| Kidney Diseases | Intensive blood pressure control is associated with a greater decrease in total kidney volume than standard blood pressure control among hypertensive patients with Autosomal dominant polycystic kidney disease (ADPKD). | [PMID25399733](https://pubmed.ncbi.nlm.nih.gov/25399733) | As compared with standard blood-pressure control, rigorous blood-pressure control was associated with a slower increase in total kidney volume, no overall change in the estimated GFR, a greater decline in the left-ventricular-mass index, and greater reduction in urinary albumin excretion. ( | support | support | 5 | 5 |
| Kidney Diseases | Intensive blood pressure control is associated with a greater decrease in total kidney volume than standard blood pressure control among hypertensive patients with Autosomal dominant polycystic kidney disease (ADPKD). | [PMID20818902](https://pubmed.ncbi.nlm.nih.gov/20818902) | In overall analyses, intensive blood-pressure control had no effect on kidney disease progression. | contradiction | contradiction | 3 | 4 |
| Kidney Diseases | Intensive blood pressure control is associated with a greater decrease in total kidney volume than standard blood pressure control among hypertensive patients with Autosomal dominant polycystic kidney disease (ADPKD). | [PMID20722556](https://pubmed.ncbi.nlm.nih.gov/20722556) | In this study, effective BP control was obtained with losartan and ramipril and LVH was found to be regressed significantly in the hypertensive patients with ADPKD. | support | support | 4 | 4 |
| Kidney Diseases | Lisinopril with telmisartan, compared to lisinopril alone, is associated with decreased rates of End-Stage Renal Disease (ESRD) among patients with Autosomal dominant polycystic kidney disease (ADPKD). | [PMID25399731](https://pubmed.ncbi.nlm.nih.gov/25399731) | There was no significant difference between the study groups in the incidence of the composite primary outcome (hazard ratio with lisinopril-telmisartan, 1.08; 95% confidence interval, 0.82 to 1.42). | contradiction | contradiction | 5 | 5 |
| Kidney Diseases | Lisinopril with telmisartan, compared to lisinopril alone, is associated with decreased rates of End-Stage Renal Disease (ESRD) among patients with Autosomal dominant polycystic kidney disease (ADPKD). | [PMID25399733](https://pubmed.ncbi.nlm.nih.gov/25399733) | In early ADPKD, the combination of lisinopril and telmisartan did not significantly alter the rate of increase in total kidney volume. | contradiction | contradiction | 5 | 5 |
| Kidney Diseases | Lisinopril with telmisartan, compared to lisinopril alone, is associated with decreased rates of End-Stage Renal Disease (ESRD) among patients with Autosomal dominant polycystic kidney disease (ADPKD). | [PMID21760473](https://pubmed.ncbi.nlm.nih.gov/21760473) | Telmisartan seems to be equivalent to enalapril in lowering BP, but telmisartan has more potent renoprotective, anti-inflammatory and antioxidative effects than enalapril in patients with hypertensive ADPKD. | support | support | 4 | 5 |
| Kidney Diseases | Lisinopril with telmisartan, compared to lisinopril alone, is associated with decreased rates of End-Stage Renal Disease (ESRD) among patients with Autosomal dominant polycystic kidney disease (ADPKD). | [PMID23195001](https://pubmed.ncbi.nlm.nih.gov/23195001) | In contrast, kidney volume increased steadily among patients on telmisartan alone both at 12 and 24 months. | support | neutral | 4 | 3 |
| Kidney Diseases | Tolvaptan is associated with decreased total kidney volume in patients with Autosomal dominant polycystic kidney disease (ADPKD). | [PMID23121377](https://pubmed.ncbi.nlm.nih.gov/23121377) | Tolvaptan, as compared with placebo, slowed the increase in total kidney volume and the decline in kidney function over a 3-year period in patients with ADPKD but was associated with a higher discontinuation rate, owing to adverse events. ( | support | support | 5 | 5 |
| Kidney Diseases | Tolvaptan is associated with decreased total kidney volume in patients with Autosomal dominant polycystic kidney disease (ADPKD). | [PMID29105594](https://pubmed.ncbi.nlm.nih.gov/29105594) | Tolvaptan resulted in a slower decline than placebo in the estimated GFR over a 1-year period in patients with later-stage ADPKD. ( | support | support | 5 | 5 |
| Kidney Diseases | Tolvaptan is associated with decreased total kidney volume in patients with Autosomal dominant polycystic kidney disease (ADPKD). | [PMID26912543](https://pubmed.ncbi.nlm.nih.gov/26912543) | ADPKD-related events were less frequent in tolvaptan recipients than in placebo recipients among those with CKD1 (hazard ratio [HR], 0.83; 95% confidence interval [95% CI], 0.70-0.98; P=0.03) and those with CKD 3 (HR, 0.71; 95% CI, 0.57-0.89; P=0.003), but not among those with CKD2 (HR, 1.02; 95% CI, 0.85-1.21; P=0.86). | support | neutral | 3 | 1 |
| Kidney Diseases | Tolvaptan is associated with decreased total kidney volume in patients with Autosomal dominant polycystic kidney disease (ADPKD). | [PMID27856088](https://pubmed.ncbi.nlm.nih.gov/27856088) | Tolvaptan decreased the incidence of kidney pain events independent of patient characteristics predisposing for kidney pain and possibly in part due to reductions in ADPKD-related complications. | support | neutral | 3 | 3 |
| Kidney Diseases | Tolvaptan is associated with decreased total kidney volume in patients with Autosomal dominant polycystic kidney disease (ADPKD). | [PMID27920153](https://pubmed.ncbi.nlm.nih.gov/27920153) | Among subjects receiving tolvaptan, those with a greater suppression of Uosm had slower renal function decline. | support | neutral | 5 | 3 |
| Hypertension | Sodium zirconium is associated with a decrease in mean serum potassium levels among patients with hyperkalemia. | [PMID25415807](https://pubmed.ncbi.nlm.nih.gov/25415807) | Patients with hyperkalemia who received ZS-9, as compared with those who received placebo, had a significant reduction in potassium levels at 48 hours, with normokalemia maintained during 12 days of maintenance therapy. ( | support | support | 5 | 5 |
| Hypertension | Sodium zirconium is associated with a decrease in mean serum potassium levels among patients with hyperkalemia. | [PMID25402495](https://pubmed.ncbi.nlm.nih.gov/25402495) | Among outpatients with hyperkalemia, open-label sodium zirconium cyclosilicate reduced serum potassium to normal levels within 48 hours; compared with placebo, all 3 doses of zirconium cyclosilicate resulted in lower potassium levels and a higher proportion of patients with normal potassium levels for up to 28 days. | support | support | 5 | 5 |
| Hypertension | Sodium zirconium is associated with a decrease in mean serum potassium levels among patients with hyperkalemia. | [PMID32149451](https://pubmed.ncbi.nlm.nih.gov/32149451) | A greater reduction in mean (Âç§SD) sK+ from baseline occurred with SZC compared with placebo at 2 hours -0.72 (Âç§0.12) versus -0.36 (Âç§0.11) mmol/L (LSM difference = -0.35 mmol/L, 95% CI = -0.68 to -0.02), respectively. | support | support | 5 | 5 |
| Hypertension | Sodium zirconium is associated with a decrease in mean serum potassium levels among patients with hyperkalemia. | [PMID25651363](https://pubmed.ncbi.nlm.nih.gov/25651363) | Thus, ZS-9 was well-tolerated in patients with stable chronic kidney disease and hyperkalemia leading to a rapid, sustained reduction in serum potassium. | support | support | 5 | 5 |
| Hypertension | Sodium zirconium is associated with a decrease in mean serum potassium levels among patients with hyperkalemia. | [PMID28421299](https://pubmed.ncbi.nlm.nih.gov/28421299) | Furthermore, SPS greatly reduced K, Mg, and NH3. | neutral | neutral | 3 | 5 |
| Hypertension | Intensive blood pressure control is associated with higher rates of all-cause mortality in nondiabetic patients with high blood pressure compared to standard blood pressure control. | [PMID34010531](https://pubmed.ncbi.nlm.nih.gov/34010531) | At a median of 3.33 years of follow-up, the rate of the primary outcome and all-cause mortality during the trial were significantly lower in the intensive-treatment group than in the standard-treatment group (rate of the primary outcome, 1.77% per year vs. 2.40% per year; hazard ratio, 0.73; 95% confidence interval [CI], 0.63 to 0.86; all-cause mortality, 1.06% per year vs. 1.41% per year; hazard ratio, 0.75; 95% CI, 0.61 to 0.92). | contradiction | contradiction | 5 | 5 |
| Hypertension | Intensive blood pressure control is associated with higher rates of all-cause mortality in nondiabetic patients with high blood pressure compared to standard blood pressure control. | [PMID24343119](https://pubmed.ncbi.nlm.nih.gov/24343119) | In conclusion, an intensive treatment aimed to lower systolic BP<130 mm Hg reduced left ventricular hypertrophy and improved clinical outcomes to a similar extent in patients with hypertension and without established cardiovascular disease. | contradiction | neutral | 4 | 3 |
| Hypertension | Intensive blood pressure control is associated with higher rates of all-cause mortality in nondiabetic patients with high blood pressure compared to standard blood pressure control. | [PMID27195814](https://pubmed.ncbi.nlm.nih.gov/27195814) | At a median follow-up of 3.14 years, there was a significantly lower rate of the primary composite outcome (102 events in the intensive treatment group vs 148 events in the standard treatment group; hazard ratio [HR], 0.66 [95% CI, 0.51-0.85]) and all-cause mortality (73 deaths vs 107 deaths, respectively; HR, 0.67 [95% CI, 0.49-0.91]). | contradiction | contradiction | 5 | 5 |
| Hypertension | Intensive blood pressure control is associated with higher rates of all-cause mortality in nondiabetic patients with high blood pressure compared to standard blood pressure control. | [PMID29021322](https://pubmed.ncbi.nlm.nih.gov/29021322) | The primary outcome hazard ratio for intensive versus standard treatment was 0.78 (95% confidence interval, 0.57-1.07) in the lowest DBP quintile (mean baseline DBP, 61Âç§5 mm Hg) and 0.74 (95% confidence interval, 0.61-0.90) in the upper 4 DBP quintiles (mean baseline DBP, 82Âç§9 mm Hg), with an interaction P value of 0.78. | contradiction | contradiction | 5 | 5 |


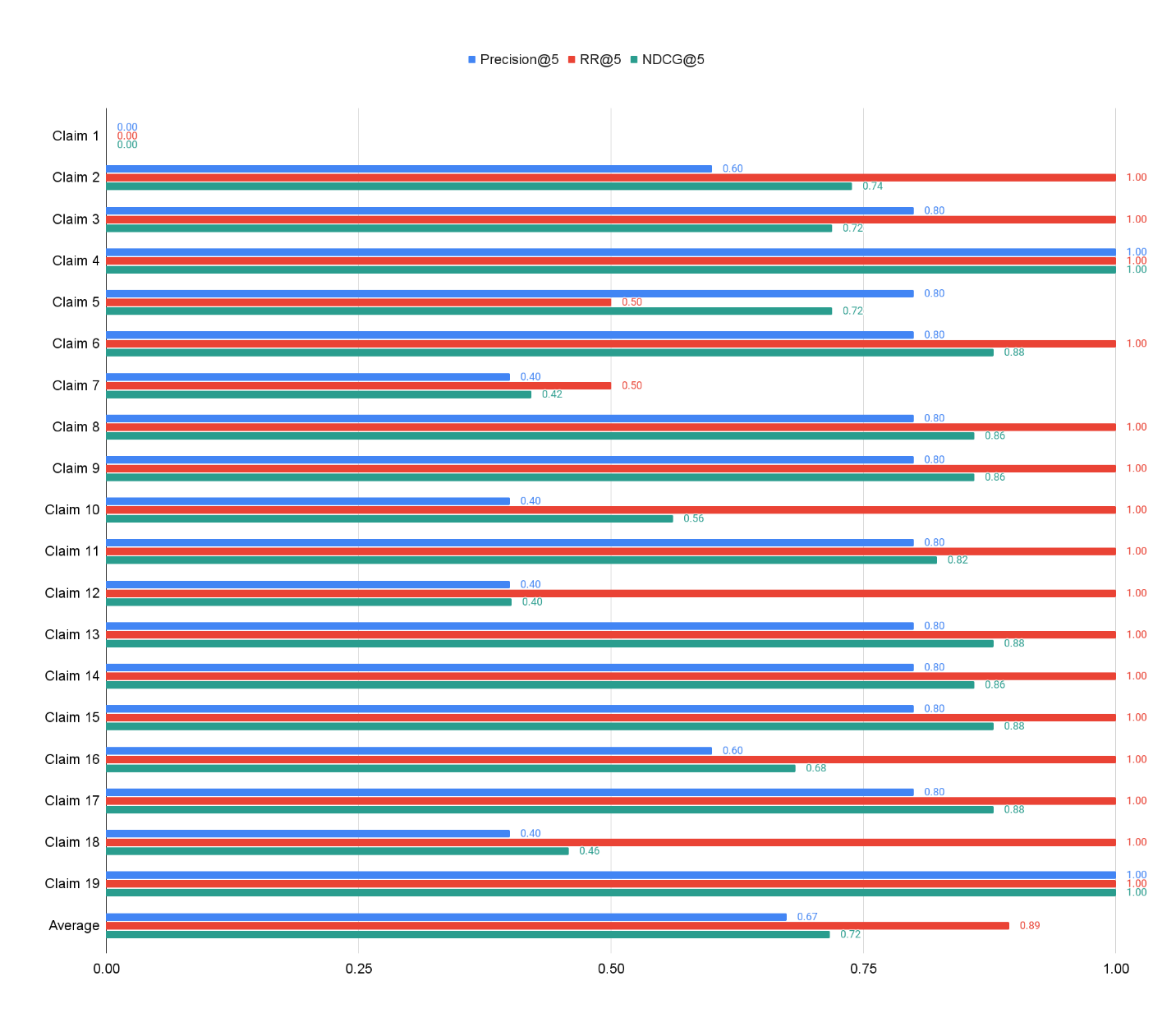


**Supplement Figure S6.** The Precision@5, RR@5, and NDCG@5 scores for each claim, and the average scores of 19 claims, on 95 abstracts retrieved by CliVER. Note that average score of RR@5 is the same as MRR@5. Precision@K is the proportion of the top-K abstracts that are relevant, and it reflects the ability of the system to retrieve relevant results among the top-K candidates. The Reciprocal Rank (RR) calculates the reciprocal of the rank at which the first relevant abstract was retrieved, within the top K abstracts if K is specified. RR is 1.0 if a relevant abstract was retrieved at rank 1 (=1/1), 0.5 if a relevant abstract was retrieved at rank 2 (=1/0.5) and so on. Mean Reciprocal Rank (MRR) is averaged RR across queries, which was calculated by averaging RR among 19 claims in our case. NDCG@K represents the extent to which a proposed ranking is in agreement with the ideal ranking, taking into account the relevance of each candidate in the top K candidates. In the calculation of NDCG score, we assigned relevance score of 1 for relevant abstracts and 0 for irrelevant abstracts with an assumption that there exists at least K relevant abstracts for any claim. Two claims (claim 4 and 19, 10.5%) achieved the perfect score of 1.0 on all three metrics. Ten claims (52.6%) achieved scores of over 0.8 on all three metrics. One claim (Claim, 15.26%). The average Precision@5 score is 0.67, the MRR@5 is 0.89, and the averaged NDCG@5 score is 0.72. The average Precision@5=0.67 indicates that CliVER found more than 3 relevant abstracts among the top-5 candidate abstracts on average. The MRR@5 score of 0.89 indicates that CliVER find the first relevant abstract within the top-2 candidates on average. The average [NDCG@5=0.72](mailto:NDCG@5=0.72) indicates that CliVER achieved a relatively good approximation (best score is 1) of ideal abstract ranking where all retrieved abstracts were relevant and sorted by their relevance in descending order.

**Supplement** **Table S7.** Comparison of the label prediction results of four different PICO-templates for claims on CoVERt. The precision, recall, and F1 score are reported, with the highest score for each metric highlighted in bold.

| Template |  |  | CoVERt |  |
| --- | --- | --- | --- | --- |
|  |  | Precision | Recall | F1 score |
| 1 |  |  |  |  |
| Support |  | 0.79 | **1.00** | 0.88 |
| Neutral |  | **1.00** | 0.89 | **0.94** |
| Refute |  | 0.93 | **0.93** | **0.93** |
| *Macro-average* |  |  |  | **0.92** |
| 2 |  |  |  |  |
| Support |  | 0.93 | 0.85 | **0.89** |
| Neutral |  | 0.89 | **1.00** | **0.94** |
| Refute |  | **0.96** | 0.82 | 0.88 |
| *Macro-average* |  |  |  | 0.90 |
| 3 |  |  |  |  |
| Support |  | 0.87 | 0.87 | 0.87 |
| Neutral |  | 0.89 | **1.00** | **0.94** |
| Refute |  | 0.94 | 0.73 | 0.82 |
| *Macro-average* |  |  |  | 0.88 |
| 4 |  |  |  |  |
| Support |  | **0.95** | 0.80 | 0.87 |
| Neutral |  | 0.85 | **1.00** | 0.92 |
| Refute |  | **0.96** | 0.77 | 0.85 |
| *Macro-average* |  |  |  | 0.88 |
